# Phylogeography, mitochondrial DNA diversity, and demographic history of geladas (*Theropithecus gelada*)

**DOI:** 10.1371/journal.pone.0202303

**Published:** 2018-08-23

**Authors:** Dietmar Zinner, Anagaw Atickem, Jacinta C. Beehner, Afework Bekele, Thore J. Bergman, Ryan Burke, Sofya Dolotovskaya, Peter J. Fashing, Spartaco Gippoliti, Sascha Knauf, Yvonne Knauf, Addisu Mekonnen, Amera Moges, Nga Nguyen, Nils Chr. Stenseth, Christian Roos

**Affiliations:** 1 Cognitive Ethology Laboratory, German Primate Center (DPZ), Leibniz Institute for Primate Research, Kellnerweg 4, Göttingen, Germany; 2 Primate Genetics Laboratory, German Primate Center (DPZ), Leibniz Institute for Primate Research, Kellnerweg 4, Göttingen, Germany; 3 Department of Psychology, University of Michigan, Ann Arbor, MI, United States of America; 4 Department of Anthropology, University of Michigan, Ann Arbor, MI, United States of America; 5 Department of Zoological Sciences, College of Natural Sciences, Addis Ababa University, Addis Ababa, Ethiopia; 6 Department of Ecology and Evolutionary Biology, University of Michigan, Ann Arbor, MI, United States of America; 7 Long-Term Ecology Laboratory, Department of Zoology, University of Oxford, Oxford, United Kingdom; 8 Department of Anthropology & Environmental Studies Program, California State University Fullerton, Fullerton, CA, United States of America; 9 Società Italiana per la Storia della Fauna “G. Altobello”, Viale Liegi 48A, Roma, Italy; 10 Work Group Neglected Tropical Diseases, Infection Biology Unit, German Primate Center, Leibniz-Institute for Primate Research, Kellnerweg 4, Göttingen, Germany; 11 Department of Animal Sciences, University of Göttingen, Burckhardtweg 2, Göttingen, Germany; 12 Centre for Ecological and Evolutionary Synthesis (CEES), Department of Biosciences, University of Oslo, Blindern, Oslo, Norway; 13 Department of Biology, College of Natural Sciences, Bahir Dar University, Bahir Dar, Ethiopia; 14 Gene Bank of Primates, German Primate Center (DPZ), Leibniz Institute for Primate Research, Kellnerweg 4, Göttingen, Germany; National Cheng Kung University, TAIWAN

## Abstract

The large-bodied, terrestrial primates in the tribe Papionini are among the most intensely studied animals in the world, yet for some members of this tribe we know comparatively little about their evolutionary history and phylogeography. Geladas (*Theropithecus gelada* Rüppell, 1835), endemic primates of the Ethiopian highlands, are largely unstudied both in genetic diversity and intrageneric phylogeny. Currently, a northern and central subspecies and one isolated southern population are recognized, of which the central is classified as Least Concern, the northern as Vulnerable, and the southern is not yet assessed. The distribution and taxonomy of the subspecies remain poorly defined. Here, we estimate the mitochondrial DNA (mtDNA) diversity and phylogenetic relationships among gelada mtDNA lineages based on samples across the entire species range. We analysed 1.7 kb-long sequences of the mtDNA genome, spanning the cytochrome *b* gene and the hypervariable region I of the D-loop, derived from 162 faecal samples. We detected five major haplogroups or clades (south, central-1, central-2, north-1, north-2) which diverged between 0.67 and 0.43 million years ago, thus suggesting a rapid radiation, resulting in largely unresolved intrageneric phylogenetic relationships. Both, the northern and central demes contain two similarly valid haplogroups, each with little or no geographic segregation among respective haplogroups. Effective population sizes of the northern and central demes decreased during and after the last glacial maximum but remained stable for the southern deme, although on a very low level. The distribution of haplogroups within the geographic ranges of the putative gelada subspecies indicates that mtDNA sequence information does not allow reliable taxonomic inferences and thus is not sufficient for solving the taxonomic rank of the three demic populations, with the possible exception of the southern population. Nevertheless, due to the genetic differences all three populations deserve conservation efforts, in particular the smallest southern population.

## Introduction

An essential precondition for effective species conservation is recognizing what exactly we need to preserve, making species delimitation and the definition of evolutionary and management units crucial for conservation planning [1–4]. Endemic mammals of the Ethiopian highlands are facing extinction risks because of intense human encroachment and habitat alteration, which lead to population fragmentation and decline (e.g., walia ibex *Capra walie*, [5]; mountain nyala *Tragelaphus buxtoni*, [6]; Ethiopian wolf *Canis simensis*, [7]; Bale monkey *Chlorocebus djamdjamensis*, [8]).

Geladas (*Theropithecus gelada* Rüppell, 1835), also endemic to the Afro-alpine grasslands of the Ethiopian Plateau, are the only extant taxon of a once species-rich genus formerly widely distributed in Africa and Eurasia [9–13]. The genus *Theropithecus* is closely related to baboons (*Papio*), kipunjis (*Rungwecebus*), and crested mangabeys (*Lophocebus*); however, the phylogenetic relationships among these genera remain uncertain [14–17]. Currently, two extant subspecies are recognized within *Theropithecus gelada–Theropithecus gelada gelada* Rüppell, 1835 and *Theropithecus gelada obscurus*, Heuglin, 1863, but their validity and geographic distributions are unclear [18–24]. Although geladas have been the subjects of several, intensive behavioural studies (e.g., [25–27]), their intra-specific phylogenetic relationships remain unresolved [28–30]. According to IUCN, *T*. *g*. *gelada* is classified as “Vulnerable” [31], whereas *T*. *g*. *obscurus* is classified as “Least Concern” [32], though these assessments were based primarily on differences in the extent of distribution in the absence of empirical data on abundance.

**Fig 1:**
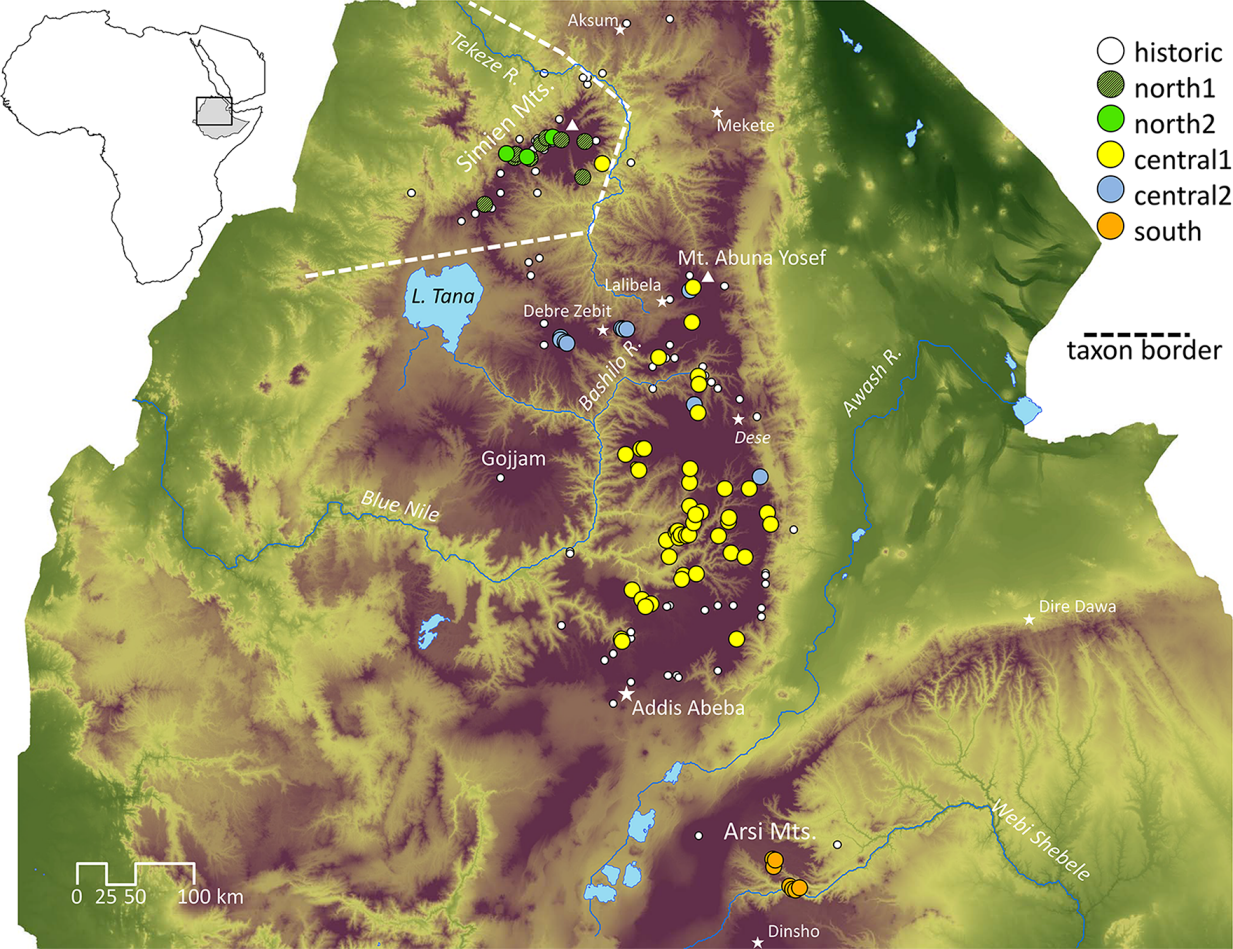
Geographic distribution of gelada sampling sites in the Ethiopian highlands. Collection of samples for this study was conducted from 2014–2016. Dashed line = proposed border between *T*. *g*. *gelada* (west of border) and *T*. *g*. *obscurus* according to [29]. White circles = historical gelada records (some of which are disputed) according to [19]; coloured circles = our sampling sites, colours indicate haplogroup affiliation. Digital Elevation Model (DEM) base map [41].

Also, recent observations conflict with historic records for several gelada populations. For example, a few of the northernmost populations reported from Tigray, which were mentioned by Yalden et al. [19] (see also Fig 1) and Gippoliti [29], appear to have gone extinct after severe droughts and many years of war (pers. obs. A. Atickem). Additionally, several authors report occurrences of geladas in Gojjam, south of Lake Tana and west of the Abbai or Blue Nile gorge [33,34]. These reports derive from a single remark by Rüppell [35], who never actually visited the province for confirmation. But, two specimens collected in 1926 by A.M. Bailey in the area between Enjiabara (Injibara)—Sakalla (Sacala) (11.020N 37.080E) and about 8 km east of Jigga (Jiga) (10.400N 37.570E) are currently housed in the Field Museum of Natural History, Chicago [29]. Our team visited Gojjam in 2015 and, despite an extensive survey, found no evidence of geladas in that region (pers. obs. R. Burke and A. Moges).

One provisional classification [29] proposes that *T*. *g*. *gelada* occurs north of Lake Tana and west of the Tekeze River (the “northern” population), whereas *T*. *g*. *obscurus* occurs south of Lake Tana and east of the Tekeze River (the “central” population; Fig 1). However, this leaves out an isolated population of geladas discovered southeast of the Rift Valley in the Arsi region along the Webi-Shebeli gorge [36]. (Note that the Arsi population of geladas had already been discovered by Neumann ([37], p. 398), who mentioned “Jellada” baboons in the Arsi region (see Fig 1)). We will refer to the Arsi geladas as the “southern” population. This southern population has not been assessed by IUCN, but owing to the small extent of suitable habitat along the gorges of the Webi Shebeli [36], it is believed to be highly threatened [38].

Morphologically, the southern population resembles the geographically distant *T*. *g*. *gelada* (northern population), rather than the geographically more proximate *T*. *g*. *obscurus* (central population) [24,29,36]. Based on the results of preliminary genetic analyses, along with geographic separation and phenotypic differences, it has been suggested that the southern Arsi population may represent a new taxon [36,39,40]; and Shotake et al. [30] tentatively named it *Theropithecus gelada arsi*. In a recent study, Shotake et al. [30] found genetic differences among the southern, northern, and central populations based on a fragment of the hypervariable region I (HVI) of the mitochondrial D-loop. Based on this limited mitochondrial sequence information and a relative limited geographic sampling (seven sites), Shotake et al. [30] reconstructed a gelada phylogeny, in which all three populations constitute monophyletic clades with the southern population representing the basal sister clade to the northern (*T*. *g*. *gelada*) and central (*T*. *g*. *obscurus*) clades. Shotake et al. [30] regarded the results of their study as sufficient to support a subspecific designation for all three gelada populations.

The aim of our study was to conduct a comprehensive study on the phylogeography of *T*. *gelada*. We were able to take advantage of new data from a country-wide survey conducted from 2014–2016 where our team obtained 174 geo-referenced gelada DNA samples with extensive representation from the “northern”, “central”, and “southern” gelada populations. We were able to generate and analyse 1.7 kb mtDNA sequence information from 162 samples. In particular, we were interested in the geographical distribution of gelada mitochondrial haplotypes, in the genetic diversity of respective demic populations and their effective population sizes, and in whether the newly available genetic information justifies any taxonomic inferences.

## Methods

### Ethical statement

Sample collection was exclusively non-invasive and complied with the laws of Ethiopia and Germany and with the guidelines of the International Primatological Society. During sampling of faecal material, no animals were harmed or disturbed.

### Sample collection

During nationwide surveys of gelada distribution and abundance between 2014 and 2016 [42] (S1 File), we non-invasively collected 174 faecal samples from wild geladas. Fresh samples were collected less than ten minutes after geladas defecated, thus minimising the risk of repeated sampling from the same individuals. Geographic coordinates of each sample were assigned at the time of collection by GPS (Fig 1, S1 Table), in addition to consecutive number, date, and broad location. Faecal samples were collected and stored following the two-step protocol of [43] and [44]. Samples were stored at ambient temperature for up to three months in the field and at -20°C upon arrival in the laboratory of the German Primate Center (DPZ).

The Ethiopian Wildlife Conservation Authority, and government officials in the following provinces/regions: North and South Shoa, North Gondar, North and South Wollo Zones, and Oromia and Tigray Regions, North Shewa Zone provided permissions to conduct this research.

### DNA extraction, sequencing

We extracted total genomic DNA using the First DNA All Tissue Kit (Gen-Ial) according to the manufacturers’ protocols, with minor modifications as outlined in [45]. After extraction, DNA concentration was measured with a NanoDrop ND-1000 spectrophotometer (Peqlab) and extracts were stored at -20°C until further processing. We amplified and sequenced a ~1800 bp-long fragment of the mitochondrial genome, spanning the complete cytochrome b gene (cytb), the tRNA genes for Threonine (tRNA-Thr) and Prolin (tRNA-Pro), as well as 462 bp of the HVI region of the D-loop, via three over-lapping PCR products to ensure that sequences were obtained even if DNA was degraded. PCR products with sizes of 604–831 bp were amplified with primers 5’-AACCATCGTTGTATTTCAAC-3’ and 5’-CGTGTAGGAATAGTAAGTG-3’, 5’-AACCTCGTCCAATGAGTC-3’ and 5’-TAAAATATAATACAGATGCTAC-3’, and 5’-CAAAGCATGATATTCCGC-3’ and 5’-GAGGTAAGAACCAGATGCCG-3’. Primers were designed on the basis of available mitochondrial genome sequences from geladas. PCR reactions were carried out in a total volume of 30 μl containing 1 U Biotherm DNA Taq polymerase (Genecraft), 1x reaction buffer, 0.16 mM of each dNTP, 0.33 μM of each primer, 0.6 mg/ml BSA and ~100 ng genomic DNA. Thermo cycling conditions comprised of 94°C for 2 min, followed by 40–50 cycles of 94°C for 1 min, 54°C for 1 min and 72°C for 1 min, followed by 72°C for 5 min. We checked PCR performance on 1% agarose gels and excised PCR products with correct size from the gel. After purification with the QIAquick Gel Extraction Kit (Qiagen), PCR products were Sanger-sequenced on an ABI 3130xL sequencer (Applied Biosystems) using the BigDye Terminator 3.1 Cycle Sequencing kit (Applied Biosystems) and the amplification primers. To avoid and check for cross-sample contamination, all working steps were carried out in separate laboratories under routine precautions. Appropriate negative controls were included into all assays and 10% of randomly selected samples were re-analysed.

Obtained sequence electropherograms were checked with 4Peaks 1.8 (nucleobytes.com/4peaks/) and sequences were assembled in SeaView 4.5.4 [46]. SeaView was also used to verify correct translation of nucleotide sequences of the protein-coding cytb gene into corresponding amino acid sequences. Sequences have been deposited in GenBank (for accession numbers see S1 Table).

### Data analyses

For phylogenetic reconstructions, we used all unique gelada haplotypes that were obtained from this study and added orthologous baboon sequences from GenBank (S1 Table). The alignment comprising 71 sequences (61 gelada and 10 baboon sequences) was generated with Muscle 3.8.31 [47] as implemented in SeaView and corrected by eye. After indels and poorly aligned positions have been removed with Gblocks 0.91b [48] using standard settings, the original alignment with 1739 bp in length was reduced to 1730 bp.

For phylogenetic tree reconstruction in IQ-Tree 1.5.2 [49] and MrBayes 3.2.6 [50], we divided the dataset into two partitions, protein-coding and non-protein-coding (1–1140, 1141–1730). For both partitions, the best-fit model of sequence evolution was determined with ModelFinder [51,52] in IQ-Tree under the Bayesian Information Criterion (BIC). The HKY+G and TPM2u+I+G models were chosen for the protein-coding and non-protein-coding partition, respectively. The maximum-likelihood (ML) tree was reconstructed in IQ-Tree with 10,000 ultrafast bootstrap (BS) replicates [53] (S1 Fig), while the Bayesian tree was obtained from a Markov Chain Monte Carlo (MCMC) run with 10 million generations, sampling every 1000 generations, in MrBayes (S2 Fig). We checked convergence of all parameters and the adequacy of a 25% burn-in by assessing the uncorrected potential scale reduction factor (PSRF) [54], as calculated by MrBayes. Posterior probabilities (PP) for nodes and a phylogram with mean branch lengths were calculated from the posterior density of trees using MrBayes. Phylogenetic trees were visualized in FigTree 1.4.2 (http://tree.bio.ed.ac.uk/software/figtree/).

We estimated divergence times using a Bayesian approach implemented in the BEAST 2.4.7 package [55]. We performed two independent analyses each with 25 million generations and tree and parameter sampling occurring every 1000 generations. For all analyses, we assumed a relaxed lognormal clock model and applied a Coalescent Constant Population prior for branching rates. Both partitions were treated separately with their respective best-fit models. To calibrate the molecular clock, we applied an exponential prior for the split between *Theropithecus* and *Papio* with an offset of 4.0 and a mean of 1.0, translating into a median of 4.69 million years ago (Ma) and a 95% highest probability density (HPD) of 4.03–7.69 Ma. The calibration point relies on the oldest known *Theropithecus* fossils with an age of ca. 4 million years [12,56], suggesting that at this time *Theropithecus* was already established and, thus, separated from *Papio*. The adequacy of a 10% burn-in and convergence of all parameters was assessed by inspecting the trace of the parameters across generations using Tracer 1.6 (http://beast.bio.ed.ac.uk/Tracer). Sampling distributions of independent runs were combined with LogCombiner 2.4.2, and trees with mean node heights were summarized with TreeAnnotator 2.4.2 using a burn-in of 10%. Trees were visualized in FigTree.

For the following analyses we used the complete gelada dataset with 162 sequences, all with 1733 bp in length. To further trace phylogenetic relatedness among gelada haplotypes, we built a haplotype network in POPART 1.7 [57] using the median-joining network algorithm [58]. To check for the most likely number of clades in the dataset, we applied Automatic Barcode Gap Discovery (ABGD) [59] and AMOVA in Arlequin 3.5.2.2 [60]. For the ABGD analyses, we used Jukes-Cantor (JC), Kimura 2-parameter (K2P) and simple distance metrics as well as different relative gap widths (X = 1.0, X = 1.5), with the other parameters at default values. For the AMOVA analysis, obtained values were statistically assessed using Akaike Information Criterion (AIC) and BIC. Haplotype and nucleotide diversities were calculated for the global dataset, for southern, northern, and central populations as well as for each of the five major clades (central-1, central-2, south, north-1, north-2) with DnaSP 5 [61].

To explore the demographic history of the three main populations, we calculated Tajima’s D [62] and Fu’s F_S_ [63] as implemented in DnaSP 5 [61]. Under the assumption of neutrality, positive D and F_S_ values suggest population contraction, whereas negative values suggest population expansion. Tajima’s D is considered more powerful at detecting older and steeper population declines, whereas Fu’s F_S_ is more sensitive in detecting more recent and shallow population contraction [64]. To further infer the demographic history of gelada populations, we assessed effective population size changes using the Bayesian Skyline Plot (BSP) method [65] as implemented in BEAST. We generated BSPs for the global population as well as for the three geographic populations (south, north, central). As substitution models, we applied the best-fit models for each dataset and partition as chosen by jModeltest 2.1.10 [66,67] (S2 Table). The analyses were performed under a strict clock model with a coalescent Bayesian Skyline prior and a substitution rate of 3.545 x 10^−8^ substitutions/site/year as determined from the divergence time calculation (see above). For each dataset, we ran two independent analyses with 25 million generations, sampling every 1000 generations and a burn-in of 10%. The results of each run were checked to ensure convergence and stationarity using Tracer. The estimated effective population size was given as N_e_ multiplied by generation time. To get the effective population size, we divided the values by a generation time of 10 years, which is similar to those in drills (*Mandrillus leucophaeus*; 10–12 years, [68]) or rhesus macaques (*Macaca mulatta*; 11 years, [69]).

## Results

**Fig 2:**
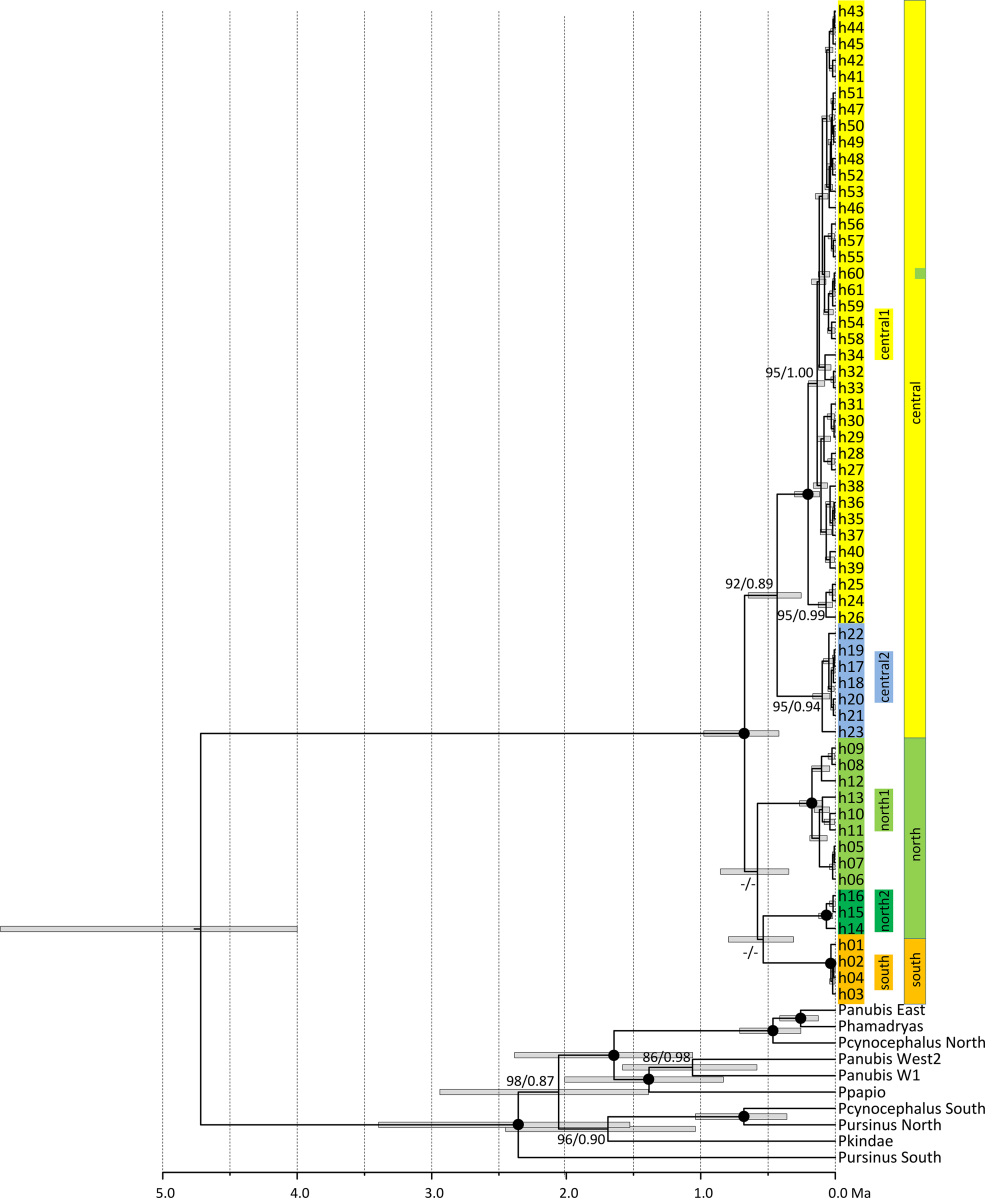
Ultrametric tree showing phylogenetic relationships and divergence times of *Theropithecus* and *Papio* mtDNA lineages. Tip labels refer to gelada and baboon haplotypes (see S1 Table). The five major gelada haplogroups (clades) and their geographic origin are indicated by colour (orange = southern, green = northern, and yellow = central; see also Fig 1). Outgroup baboon haplogroups are labelled according to [70,71]. Node labels refer to ML BS and Bayesian PP support values (black circles: BS > 98%; PP > 0.98). The time scale below the tree indicates million years ago. Haplogroup designation and geographic origin of gelada samples coincide with only one exception: one sample found in the Simien Mountains (northern population) had a mtDNA haplotype which was identical to haplotype 60 belonging to the central-1 clade.

**Fig 3:**
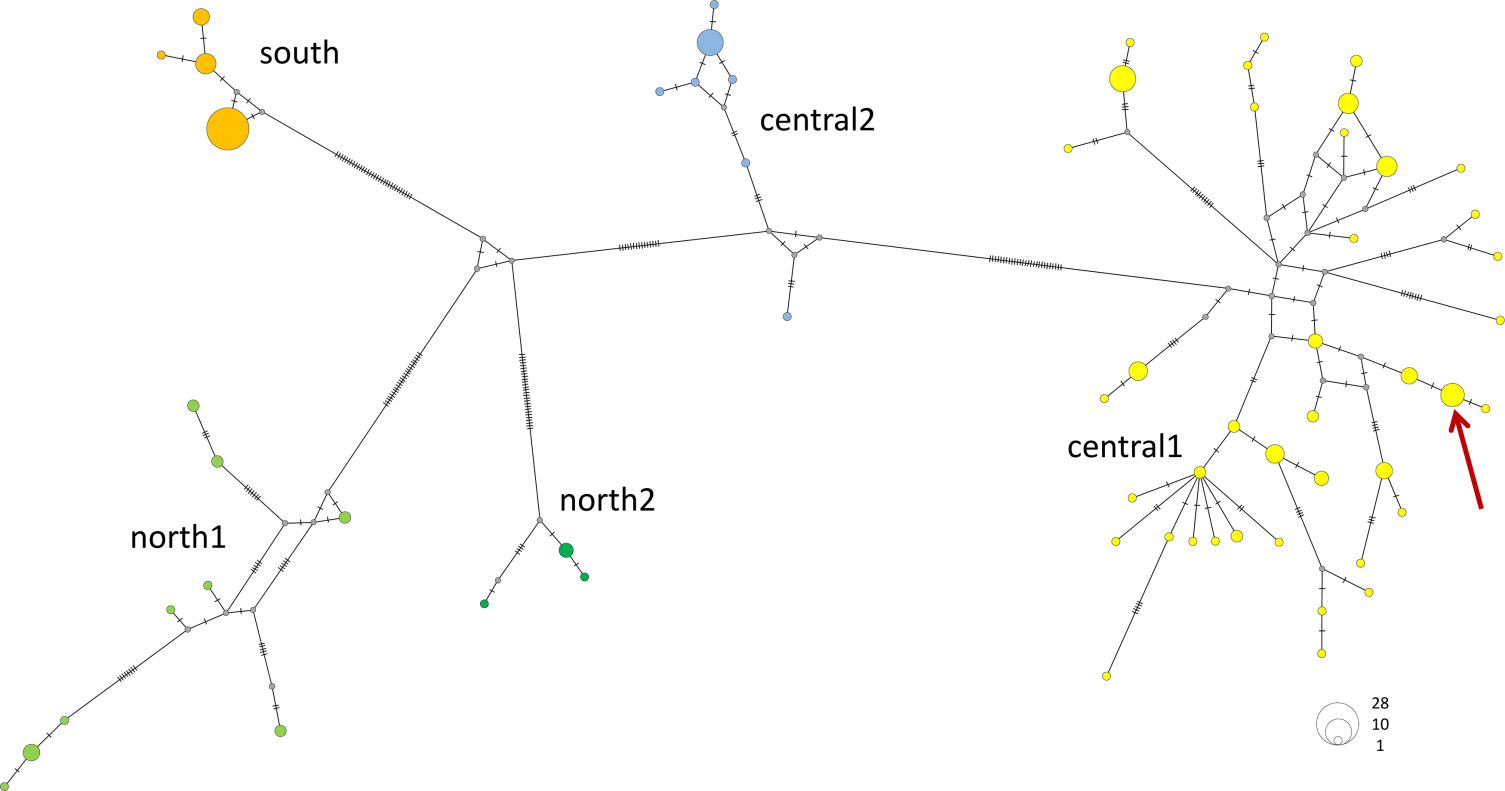
Median-joining mtDNA haplotype network of geladas (colours of haplotypes are the same as in Figs 1 and 2). The red arrow indicates haplotype 60 which contains one sample from the northern population (close to Simien Mountains). Number of hatch marks refers to the number of mutations. Numbers of identical haplotypes are indicated by vertex-size.

We successfully retrieved sequences from 162 (out of 174) faecal samples and found a total of 61 haplotypes (S1 Table). According to phylogenetic tree reconstructions, geladas segregated into five major mtDNA clades (south, north-1, north-2, central-1 and central-2; Fig 2). This 5-clade model also appeared as the best model from the haplotype network (Fig 3) and the AMOVA as it received the lowest AIC and BIC values compared to models with 3, 4, or 6 clades (S3 and S4 Tables). Likewise, the ABGD analyses using different parameter combinations revealed five clades (S5 Table). The south clade contained individuals from the Arsi region, south of the Rift Valley. Although we obtained 39 sequences from the Arsi region, we observed only four haplotypes (Table 1). The two north clades contained 16 (north-1) and five sequences (north-2), and nine and three haplotypes, respectively. Both north clades consist mainly of samples from the Simien Mountains. The central-1 clade was the largest clade with 86 sequences and 38 haplotypes, whereas clade central-2 consisted of 16 sequences and seven haplotypes. Haplogroup designation and geographic origin coincided largely with only one exception: one sample found in the Simien Mountains (northern population) had a mtDNA haplotype which was identical to haplotype 60 belonging to clade central-1 (Fig 2, S1 Table).

**Table 1:** Population genetic parameters for gelada mitochondrial clades.

demes	individuals	nucleotide sites	polymorphic sites (S)	haplotypes	haplotype diversity (*h*)*	nucleotide diversity (π)*
global	162	1733	147	61	0.954 ± 0.009	0.02195 ± 0.0007
southern	39	1733	4	4	0.462 ± 0.087	0.00062 ± 0.0001
northern	21	1733	63	12	0.938 ± 0.030	0.01402 ± 0.0017
north-1	16	1733	34	9	0.917 ± 0.046	0.00732 ± 0.0006
north-2	5	1733	7	3	0.700 ± 0.218	0.00162 ± 0.0008
central	102	1733	86	45	0.964 ± 0.007	0.01011 ± 0.0008
central-1	86	1733	60	38	0.962 ± 0.008	0.00585 ± 0.0003
central-2	16	1733	11	7	0.625 ± 0.139	0.00108 ± 0.0004

* ± SD

The two north clades did not reveal any geographic structure. Haplotypes of both clades were found admixed within the sampling range. For the two central clades, a slight geographic pattern was observed; samples from the central-2 clade were found predominantly in the northern part of the range of the central clades. However, both clades intergraded spatially (Fig 1).

Monophyly of all five clades was strongly supported, but the phylogenetic relationships among them remained largely unresolved (S1 and S2 Figs). According to our ultrametric tree (Fig 2), divergences among the five clades occurred in a relatively short time period of 250,000 years (S6 Table). At 0.67 Ma (95% HPD 0.42–0.98 Ma) the southern and northern lineages split from the central lineages, followed by the split between the combined south and north-2 clades from the north-1 clade at 0.58 Ma (95% HPD 0.35–0.85 Ma). At roughly the same time (0.54 Ma, 95% HPD 0.31–0.79 Ma), the north-2 clade split from the south clade, followed by the split between the two central clades 0.43 Ma (95% HPD 0.25–0.65 Ma). In contrast, the diversification of the major baboon mtDNA lineages started already around 2.36 Ma (split off of the southern *Papio ursinus*) and was almost completed prior to the first divergence of the gelada lineage (0.67 Ma).

Haplotype diversity (*h*) and nucleotide diversity (π) for the various gelada demes are summarized in Table 1. The haplotype diversity for the southern population (*h* = 0.462) is roughly half of that estimated for the northern (*h* = 0.938) and central populations (*h* = 0.964). For all three populations, nucleotide diversities (central: π = 0.0101, northern: π = 0.0140, southern: π = 0.0006) are relatively low, especially for the southern population. The haplotype and nucleotide diversities of the central and northern populations decrease, if we divide them into their respective two clades. However, the southern deme remains the one with the lowest genetic diversity.

The non-significant values of the neutrality tests for the central and southern demes (Table 2) indicate no departure from the null hypothesis of demographic stability. However, the significant positive values of Fu's F_S_ for the global gelada population and the northern deme indicate a recent and shallow population decline.

**Table 2:** Neutrality tests for the global gelada population and the northern, central and southern gelada demes.

	global	north	central	south
No. of individuals	162	21	102	39
No. of polymorphic sites (S)	147	63	86	4
Fu's F_S_ (*P* value)	1.8490 (< 0.05)	1.9041 (< 0.02)	0.1673 (>0.10)	0.0862 (> 0.10)
Tajima's D (*P* value)	1.4848 (> 0.10)	1.5566 (> 0.10)	0.1918 (> 0.10)	0.3184 (> 0.10)

**Fig 4:**
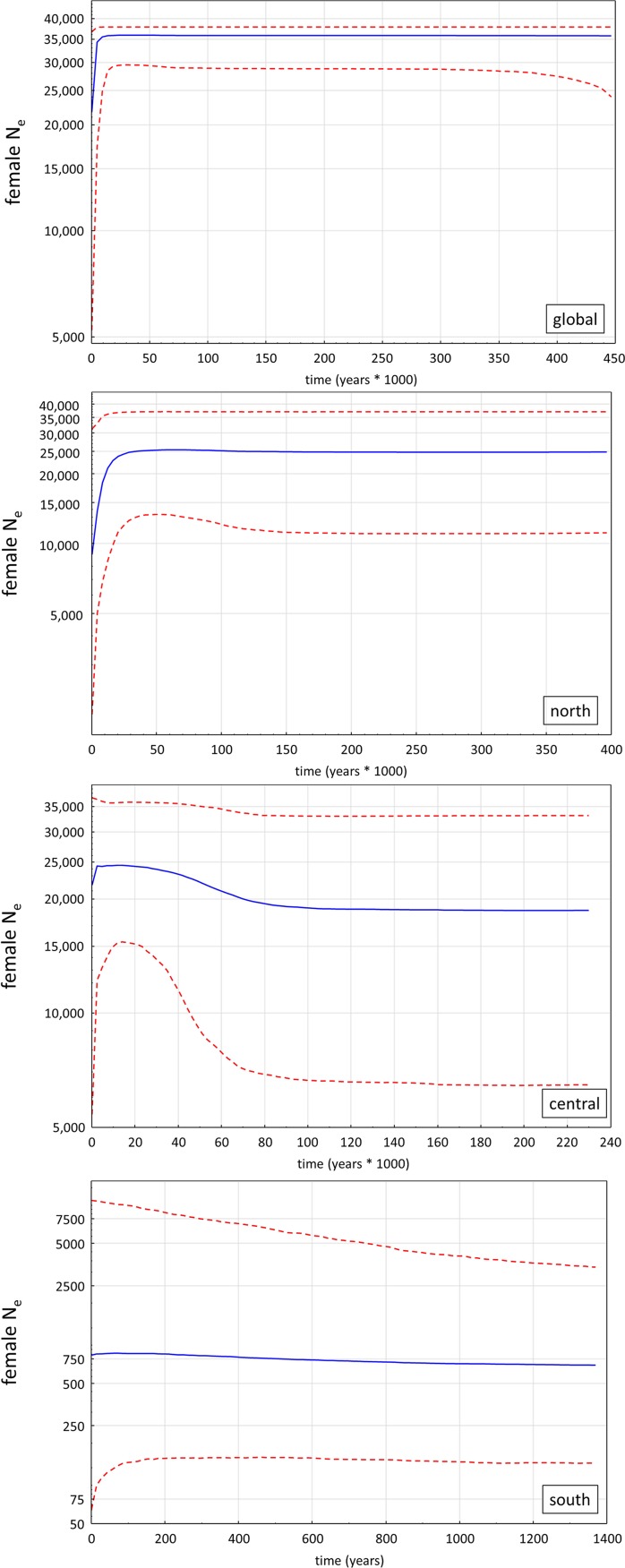
Bayesian skyline plots (BSPs) of the global gelada population and the three demes (north, central and south). The y-axes show respective effective population sizes of females (N_e_) in a log scale. Blue lines denote the median effective population sizes, red broken lines indicate respective 95% HPD limits.

The recent population declines are also obvious in our BSPs. They suggest that the global gelada population experienced a prolonged period of demographic stability (female N_e_ ~36,000), before it started to decline around 10,000 years ago to the current female N_e_ of ~22,000 (Fig 4). A similar population trend is obvious for the northern deme. Here, the population dropped from N_e_ ~25,000 to ~9,000 females. However, this decline started already around 22,000 years ago. The central and southern demes show a different pattern. The central population increased around 80,000 years ago to the maximum of almost ~25,000 females, before it decreased slightly to its current N_e_ of ~22,000 females. (Note that these values are medians with respective 95% HPD limits, which explains why the current global and central N_e_s are similar, although the central population is only a subpopulation of the global population). For the southern deme, the BSP suggests that at least within the last 1500 years the population remained stable but on a very low level of only ~750 females.

## Discussion

### Phylogeny

Our study revealed five major mtDNA clades that correspond to three distribution ranges defined as northern, central, and southern; and hence suggest a demic pattern. Results of our study are largely in agreement with [30]. However, as expected from our extended geographical sampling and application of considerably more genetic information, our results provide higher phylogenetic and geographical resolutions.

Our south clade corresponds to the Arsi region clade of Shotake et al. [30]. In contrast to [30], we found further differentiation of the northern and central populations into two clades each. The respective two north clades correspond to Shotake’s “Simien Mountains” clade. These clades do not exhibit a clear demic pattern with haplotypes of both clades occurring within the Simien Mountains. The deep divergence among the north clades is interesting because there is at least one historical report of a second gelada taxon from the region of the Simien Mountains, *Theropithecus gelada senex* Pucheran, 1857. The type specimen was collected by the German botanist W.G. Schimper near Noari, east of Ras Dashen in the Simien Mountains [72]. *T*. *g*. *senex* was later classified as a synonym of *T*. *g*. *gelada*. However, if there have been historically two morphologically different gelada taxa, which came into secondary contact with subsequent genetic exchange, the contemporary Simien gelada population might still possess genetic signals of the two taxa. This would explain why different morphotypes might appear from time to time and could have been classified (at least in one case) as a second taxon, *T*. *g*. *senex*. An alternative explanation for the occurrence of the “senex” morphotype in the Noari region could be hybridization among *T*. *g*. *gelada* and *T*. *g*. *obscurus*. An indication for a hybrid origin of the “senex” type could be the occurrence of a central clade haplotype (*T*. *g*. *obscurus*) in the range of the north clade (*T*. *g*. *gelada*). The respective sample was collected in the Noari region, close to the type locality of “senex”. A genetic analysis of the type series of “senex” stored in the Strasbourg Natural History Museum would probably help to shed some light on the phylogenetic relationship of this presumed taxon.

Whether one of the northern lineages was historically also present in Tigray and Eritrea is not clear because geladas have most likely been exterminated from their former northern range (Tigray: AA, RB pers. comm.; Eritrea DZ pers. comm.). However, survey effort in Tigray has been relatively minimal, so it cannot be ruled out that small isolated populations still exist.

Our two central clades correspond largely to Shotake’s “Blue Nile river basin” clade. Similar to our two north clades, the central clades show geographical admixture, with a tendency for central-2 clade occurring mainly in the northwest of the central range. In addition to [30], we discovered that the central clades extend north across the Bashilo River into the area of Mt. Abuna Yosef. Given the reported high phenotypic diversity known among “*obscurus”* [29], it would be worthwhile to further investigate whether parts of the phenotypic variation can be explained by haplogroup membership.

In general, we found genetic diversity distributed into three demes with one exception. One sample with a central haplotype (h60) was found within the geographic range of the northern haplotypes, east of the Simien Mountains but west of the Tekeze River. Identical haplotypes have been found at least 350 km south. With the currently available information, we can only speculate about possible causes for this geographical mismatch. It can be a relict of a former partly overlapping distribution of the central and north clades, or it is possible that humans have transferred single individuals from one area to another. It is known at least for infant baboons in Eritrea that they are kept as pets and transported over large distances, where they are occasionally released into wild populations (pers. observation DZ).

Although the five clades are well supported, their branching pattern is not well-resolved (S1 and S2 Figs). However, our phylogenetic reconstruction does also not suggest clade relationships that correspond to their respective parapatric provenances, i.e., northern haplotypes are probably more closely related to the distant southern haplotypes instead of the geographically closer central haplotypes. Similarly, Shotake et al. [30] detected a closer relationship of the two geographically most-distant clades (Arsi and Simien) than either of the two to the geographically closer “Blue Nile river basin” clade. The unresolved phylogenetic relationships among our five clades also prevent further phylogeographic analyses, such as reconstruction of ancestral areas.

According to our divergence time estimations, the first split between the southern + northern and central lineages occurred around 0.67 Ma and the divergence into the five major mtDNA lineages was completed around 0.43 Ma. Our estimated divergence ages are slightly older than those reported by [30], who estimated the first split around 0.4 Ma, although the lower limits of our estimated HPDs are in a similar range (S5 Table). Compared to the divergences of the main mtDNA lineages in *Papio*, divergence ages within geladas are relatively recent (Fig 2). On the other hand, the relatively deep divergence ages among the respective north and central clades suggest that they experienced a period of geographic isolation and independent evolutionary history before they merged again geographically. Our divergence age estimates suggest that the split of the main five gelada mtDNA lineages was not caused by climate changes during the last three or four glacial cycles. These periods are younger than our estimates (< 500,000 years [73]).

### Taxonomic implications

The taxonomy of geladas is disputed [29,30,74], and our results contribute only marginally to its resolution. Based on the biogeographical patterns, genetic differences, and divergence times, Shotake et al. [30] suggest a subspecific (if not specific) rank for the three main populations. In contrast, our results do not support a taxon delineation based on mtDNA information. With the exception of the south clade, there is incomplete geographic segregation between the respective two north and two central clades. Furthermore, one should be cautious using the similarity of divergence ages between Ethiopian *Papio anubis* and *Papio hamadryas* on the one hand, and those of the central and north gelada clades on the other hand, as an argument for subspecific status of the two gelada populations, as suggested by [30]. In *Papio*, mtDNA markers were unable to resolve the taxonomy because of hybridization and introgression [70,71,75,76]. Since *P*. *anubis* most likely introgressed *P*. *hamadryas* populations of Eritrea and Ethiopia, they carry mitochondria derived from *P*. *hamadryas*. In fact, the divergence age between the mtDNA lineages of the two Ethiopian baboon species refers to an intra-taxon–not an inter-taxon–divergence and, thus, is not suitable to be used as a means to delimit subspecies. In many cases, mtDNA markers alone are sufficient to detect cryptic species and to delimit taxonomic groups, but in other cases, this is not possible [3]. However, given the particular biogeographical and phylogenetic position of the southern population, an allocation to the subspecies *T*. *g*. *arsi* is indicated, but more genetic (nuclear markers, e.g. microsatellites, single nucleotide polymorphisms, or whole genomes) and morphological information are necessary for a proper internal taxonomic revision of *Theropithecus*.

### Genetic diversity and conservation

As reported by [30] and also found in our study, intraspecific genetic variation is generally low in geladas. In particular, the southern population seems to be genetically impoverished, which might be an effect of the overall small population size in combination with genetic drift or a population bottleneck. Haplotype diversity is comparable to estimates found in *Rhinopithecus* although nucleotide diversity in geladas is smaller than in this Asian colobine genus [77]. Compared to African hamadryas baboons (*P*. *hamadryas*; [78], genetic diversity in geladas is significantly lower (n = 149; haplotype diversity: *h* ± SD = 0.983 ± 0.003, p < 0.001; nucleotide diversity: π ± SD = 0.04251 ± 0.00088, p < 0.001).

The relatively low genetic diversity, in the southern population in particular, at first, might tempt conservation managers to increase genetic diversity by translocation of extra-demic geladas. However, such genetic rescue by translocation should be avoided here, since the preservation of the different evolutionary lineages of geladas will be undermined and any local adaptation would likely be lost, with possible negative consequences for survival in this marginal habitat [79–81]. The low genetic diversity of the southern population translates into an extremely low female effective population size (Fig 4). Female effective population sizes of the central and northern populations are considerable higher, however, they experienced a sharp decrease after the last glacial maximum.

The combination of relatively low genetic diversity and the apparent extirpation of several local populations over the last century, a trend that is suspected or confirmed in another endemic species to the Afromontane region–the endangered Ethiopian Wolf *Canis simiensis* [82]–supports the urgent need for conservation action of at least the southern gelada population and its ecosystem in general. The Afro-alpine grasslands in Ethiopia, on which geladas and other endemic species depend, is not only threatened by anthropogenic habitat alteration, but will most likely also shrink as a result of global warming [83,84]. At the very least and as a first step towards effective conservation of geladas, their three main demes should be regarded as independent conservation management units.

## Supporting information

S1 Fig ML tree showing phylogenetic relationships among gelada and baboon haplotypes. Numbers at nodes refer to bootstrap values in %.
(PDF)

S2 Fig Bayesian tree showing phylogenetic relationships among gelada and baboon haplotypes. Numbers at nodes refer to posterior probabilities.
(PDF)

S1 Table Geographic provenance of gelada samples (decimal degrees), their mtDNA haplotypes (h-type), haplogroups (h-group) and GenBank Accession Numbers. * Sample from northern range but with central haplotype.
(PDF)

S2 Table Best-fit models for protein-coding and non-protein-coding partitions for various gelada populations as obtained from jModeltest for the Bayesian skyline plots (BSPs). (PDF)

S3 Table AMOVA results. (PDF)

S4 Table Selection for the “best” population model. (PDF)

S5 Table Results of the Automatic Barcode Gap Discovery (ABGD) analyses. (PDF)

S6 Table Divergence age estimates (Ma) and respective 95% Highest Posterior Density (HPD) intervals. (PDF)

S1 File Nguyen N, Fashing PJ, Burke RJ. Determining the conservation status of gelada monkeys: Distribution, abundance and phylogenetic relationships of *Theropithecus gelada* across the Ethiopian Highlands. 2016; Unpubl. Final report to Margot Marsh Biodiversity Foundation.
(PDF)
