# Supplementary material for: Phylogeography, mitochondrial DNA diversity, and demographic history of geladas (Theropithecus gelada)
Source: PLoS One. 2018 Aug 23;13(8):e0202303. doi: 10.1371/journal.pone.0202303 (PMC6107150; doi:10.1371/journal.pone.0202303)
Supplement: S2 Fig — Numbers at nodes refer to posterior probabilities. (PDF) [file pone.0202303.s002.pdf]

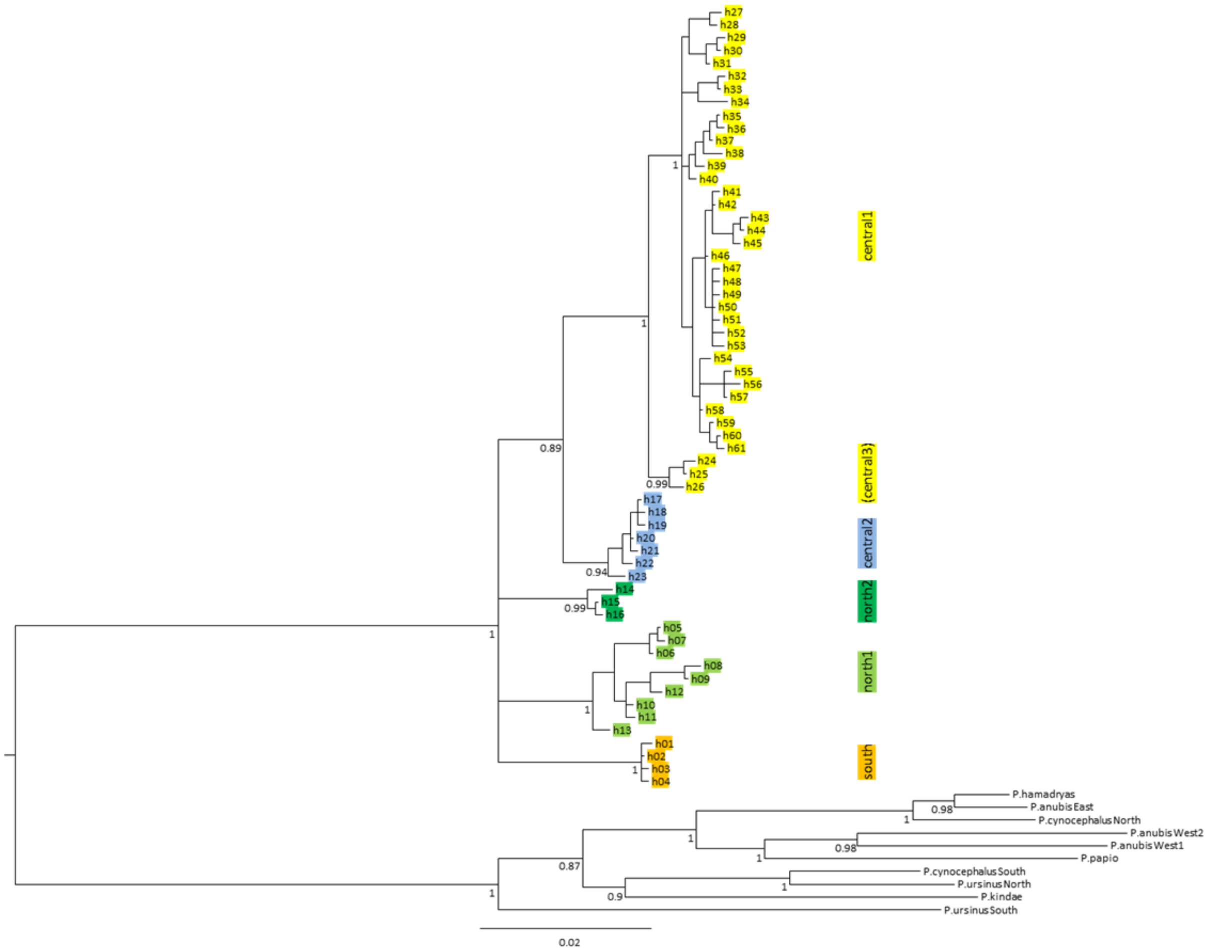

S2 Fig. Bayesian tree showing phylogenetic relationships among gelada and baboon haplotypes. Numbers at nodes refer to posterior probabilities.
