# Supplementary material for: Phylogeography, mitochondrial DNA diversity, and demographic history of geladas (Theropithecus gelada)
Source: PLoS One. 2018 Aug 23;13(8):e0202303. doi: 10.1371/journal.pone.0202303 (PMC6107150; doi:10.1371/journal.pone.0202303)
Supplement: S1 Table — * Sample from northern range but with central haplotype. (PDF) [file pone.0202303.s003.pdf]

S1 Table. Geographic provenance of gelada samples (decimal degrees), their mtDNA haplotypes (h-type), haplogroups (h-group) and haplotype GenBank Accession Numbers. * Sample from northern range but with central haplotype.

Sample ID	region	h-type	h-group	latitude	longitude	Acc. No.
TG002	Arsi	1	south	7.703217	39.817550	MH634017
TG019	Arsi	1	south	7.696067	39.817353	
TG020	Arsi	1	south	7.704215	39.818511	
TG028	Arsi	1	south	7.697065	39.816058	
TG062	Arsi	1	south	7.695133	39.812400	
TG063	Arsi	1	south	7.739117	39.836167	
TG064	Arsi	1	south	7.749650	39.815467	
TG065	Arsi	1	south	7.746700	39.814083	
TG079	Arsi	1	south	7.696582	39.817762	
TG080	Arsi	1	south	7.697346	39.815814	
TG081	Arsi	1	south	7.697684	39.813783	
TG083	Arsi	1	south	7.702782	39.815724	
TG084	Arsi	1	south	7.691811	39.825015	
TG085	Arsi	1	south	7.691188	39.824134	
TG086	Arsi	1	south	7.690380	39.820923	
TG087	Arsi	1	south	7.691517	39.822394	
TG088	Arsi	1	south	7.696558	39.816138	
TG091	Arsi	1	south	7.693582	39.825562	
TG092	Arsi	1	south	7.699043	39.812643	
TG093	Arsi	1	south	7.695001	39.816661	
TG094	Arsi	1	south	7.696318	39.818595	
TG095	Arsi	1	south	7.695205	39.823553	
TG096	Arsi	1	south	7.693248	39.825525	
TG105	Arsi	1	south	7.703111	39.813603	
TG106	Arsi	1	south	7.696121	39.817534	
TG107	Arsi	1	south	7.693548	39.824483	
TG108	Arsi	1	south	7.693548	39.824483	
TG109	Arsi	1	south	7.693548	39.824483	
TG007	Arsi	2	south	7.500550	39.991950	MH634018
TG052	Arsi	2	south	7.500917	39.991600	
TG053	Arsi	2	south	7.500433	39.992117	
TG057	Arsi	2	south	7.516167	39.967533	
TG059	Arsi	2	south	7.539133	39.944733	
Ars08	Arsi	2	south	7.502335	39.989432	
TG058	Arsi	3	south	7.539467	39.945567	MH634019
TG055	Arsi	4	south	7.509000	39.991167	MH634020
TG056	Arsi	4	south	7.509833	39.990650	
TG060	Arsi	4	south	7.526800	40.021167	
TG061	Arsi	4	south	7.696102	39.818259	
TG045	N. Gondar	5	north-1	13.131680	37.931390	MH634021
TG047	N. Gondar	5	north-1	13.131900	37.932280	
TG048	N. Gondar	5	north-1	13.128790	37.943650	
TG066	N. Gondar	5	north-1	13.121380	37.933230	
TG068	N. Gondar	6	north-1	13.231450	38.039550	MH634022
TG103	N. Gondar	7	north-1	13.129000	37.943410	MH634023
TG051	N. Gondar	8	north-1	13.153230	37.844510	MH634024
TG137	N. Gondar	8	north-1	13.143190	37.844230	
NGon_104	Wag Hemira	9	north-1	12.977109	38.357779	MH634025
NGon_106	Wag Hemira	9	north-1	12.977109	38.357779	
Smn01	N. Gondar	10	north-1	13.262234	38.192206	MH634026
Smn04	N. Gondar	11	north-1	13.277246	38.080997	MH634027
TG044	N. Gondar	12	north-1	13.126360	37.834810	MH634028
TG050	N. Gondar	12	north-1	12.769030	37.607040	
NGon_056	N. Gondar	13	north-1	13.250211	38.373131	MH634029
NGon_100	N. Gondar	13	north-1	13.250211	38.373131	
TG046	N. Gondar	14	north-2	13.121230	37.933710	MH634030
TG043	N. Gondar	15	north-2	13.131680	37.931390	MH634031

TG067	N. Gondar	15	north-2	13.153230	37.844510	
Smn07	N. Gondar	15	north-2	13.283909	38.127183	
TG004	N. Gondar	16	north-2	13.121230	37.933710	MH634032
TG030	N. Wollo	17	central-2	11.816670	38.657860	MH634033
TG110	N. Wollo	17	central-2	11.808840	38.686060	
TG120	N. Wollo	17	central-2	11.811000	38.695720	
SGon_048	S. Gondar	17	central-2	11.702353	38.237365	
SGon_049	S. Gondar	17	central-2	11.702353	38.237365	
SGon_050	S. Gondar	17	central-2	11.702381	38.237420	
SGon_051	S. Gondar	17	central-2	11.702372	38.237438	
SGon_052	S. Gondar	17	central-2	11.702435	38.237356	
SGon_053	S. Gondar	17	central-2	11.702363	38.237466	
SGon_054	S. Gondar	17	central-2	11.730750	38.188872	
SGon_005	S. Gondar	18	central-2	11.730841	38.189019	MH634034
SGon_004	S. Gondar	19	central-2	11.730471	38.189360	MH634035
TG077	N. Shoa	20	central-2	10.678740	39.719030	MH634036
TG119	N. Wollo	21	central-2	11.812570	38.670560	MH634037
Abn01	N. Wollo	22	central-2	12.117124	39.186450	MH634038
SWol_023	S. Wollo	23	central-2	11.232203	39.215138	MH634039
TG033	N. Shoa	24	central-1	9.432482	38.658693	MH634040
TG024	N. Shoa	25	central-1	9.432482	38.658693	MH634041
TG025	N. Shoa	25	central-1	9.434728	38.653225	
TG034	N. Shoa	25	central-1	9.434728	38.653225	
TG121	N. Shoa	25	central-1	9.434728	38.653225	
TG122	N. Shoa	25	central-1	9.434728	38.653225	
TG124	N. Shoa	25	central-1	9.434728	38.653225	
TG126	N. Shoa	25	central-1	9.434728	38.653225	
TG129	N. Shoa	25	central-1	9.434728	38.653225	
TG131	N. Shoa	25	central-1	9.434728	38.653225	
DeL1609	N. Shoa	25	central-1	9.703301	38.880941	
TG133	N. Shoa	26	central-1	9.924010	39.129380	MH634042
TG078	N. Shoa	27	central-1	10.094470	39.495600	MH634043
TG074	N. Shoa	28	central-1	10.317580	39.804930	MH634044
Zur_068	N. Wollo	29	central-1	12.130486	39.204684	MH634045
SWol_016	N. Shoa	30	central-1	10.634220	39.177140	MH634046
SWol_032	S. Wollo	31	central-1	10.586670	39.447272	MH634047
TG003	N. Shoa	32	central-1	10.365550	39.480190	MH634048
TG006	N. Shoa	33	central-1	10.313260	39.799470	MH634049
TG069	N. Shoa	33	central-1	10.326640	39.803830	
TG070	N. Shoa	33	central-1	10.326660	39.803770	
TG071	N. Shoa	33	central-1	10.327440	39.804900	
TG076	N. Shoa	33	central-1	10.337000	39.477100	
SWol_021	S. Wollo	34	central-1	11.169830	39.240765	MH634050
TG013	S. Wollo	35	central-1	10.729859	38.789249	MH634051
TG014	S. Wollo	35	central-1	10.731104	38.783797	
TG026	S. Wollo	35	central-1	10.730526	38.785590	
TG111	S. Wollo	35	central-1	10.731321	38.784492	
TG112	S. Wollo	35	central-1	10.730996	38.785188	
TG115	S. Wollo	35	central-1	10.734442	38.785642	
TG012	S. Wollo	36	central-1	10.892899	38.821098	MH634052
TG118	S. Wollo	36	central-1	10.891400	38.809970	
TG027	S. Wollo	37	central-1	10.892862	38.820695	MH634053
TG032	S. Wollo	37	central-1	10.408524	39.264562	
TG036	S. Wollo	37	central-1	10.325930	39.208310	
TG113	S. Wollo	37	central-1	10.890296	38.809433	
TG114	S. Wollo	37	central-1	10.894209	38.804187	
TG116	S. Wollo	37	central-1	10.892718	38.820613	
NWol_025	N. Wollo	38	central-1	11.594332	38.941212	MH634054
WOL07	S. Wollo	39	central-1	10.850275	38.686786	MH634055
TG117	S. Wollo	40	central-1	10.893369	38.805642	MH634056
TG140	N. Shoa	41	central-1	10.262383	39.086452	MH634057
TG141	N. Shoa	41	central-1	10.262383	39.086452	
TG142	N. Shoa	41	central-1	10.262383	39.086452	
TG001	N. Shoa	42	central-1	10.063080	39.603140	MH634058
TG011	N. Shoa	42	central-1	9.737423	38.812659	

TG016	N. Shoa	42	central-1	10.190600	39.002054	
TG147	N. Shoa	42	central-1	10.211667	39.096500	
TG148	N. Shoa	42	central-1	10.211667	39.096500	
Gua1610	N. Shoa	43	central-1	10.313260	39.799470	MH634059
TG072	N. Shoa	44	central-1	10.317580	39.804930	MH634060
GUS12	N. Shoa	45	central-1	10.402772	39.772878	MH634061
TG035	N. Shoa	46	central-1	10.225650	39.122140	MH634062
TG139	N. Shoa	46	central-1	10.587705	39.634344	
TG097	N. Shoa	47	central-1	10.223450	39.399720	MH634063
SWol_020	S. Wollo	48	central-1	11.450912	39.244585	MH634064
TG143	N. Shoa	49	central-1	10.232560	39.171980	MH634065
TG144	N. Shoa	49	central-1	10.226560	39.153490	
TG005	N. Wollo	50	central-1	11.863720	39.196170	MH634066
SWol_018	S. Wollo	50	central-1	10.451690	39.176899	
SWol_019	S. Wollo	51	central-1	11.386291	39.249103	MH634067
SWol_017	S. Wollo	52	central-1	10.634157	39.176948	MH634068
TG009	N. Shoa	53	central-1	9.811476	38.736883	MH634069
TG021	N. Shoa	54	central-1	9.703301	38.880941	MH634070
TG029	N. Shoa	54	central-1	9.703301	38.880941	
TG149	N. Shoa	55	central-1	10.211667	39.096500	MH634071
SWol_015	S. Wollo	56	central-1	10.740342	39.181793	MH634072
TG017	S. Wollo	57	central-1	10.409266	39.264435	MH634073
TG018	S. Wollo	57	central-1	10.408524	39.264562	
TG022	S. Wollo	57	central-1	10.409266	39.264435	
TG031	S. Wollo	57	central-1	10.888970	38.813670	
TG134	N. Shoa	58	central-1	9.924010	39.129380	MH634074
TG135	N. Shoa	58	central-1	9.924010	39.129380	
TG136	N. Shoa	58	central-1	9.924010	39.129380	
TG010	N. Shoa	59	central-1	9.811675	38.736983	MH634075
TG098	N. Shoa	59	central-1	9.434530	39.538910	
TG104	N. Shoa	59	central-1	9.434960	39.539400	
TG132	N. Shoa	59	central-1	9.924010	39.129380	
TG008	N. Shoa	60	central-1	10.066174	39.020854	MH634076
TG015	N. Shoa	60	central-1	10.251062	39.074915	
TG049*	Wag Hemira	60	central-1	13.081910	38.508000	
TG099	N. Shoa	60	central-1	9.434890	39.539340	
TG101	N. Shoa	60	central-1	9.934660	39.231030	
TG138	N. Shoa	60	central-1	10.212790	39.090420	
TG145	N. Shoa	60	central-1	10.233470	39.113140	
TG100	N. Shoa	61	central-1	9.924010	39.129380	MH634077
Papio ursinus (S)						JX946204
P. ursinus (N)						JX946205
P. cynocephalus (S)						JX946200
P. cynocephalus (N)						JX946199
P. kindae						JX946202
P. papio						JX946203
P. anubis (W1)						JX946197
P. anubis (W2)						JX946198
P. anubis (E)						JX946196
P. hamadryas						JX946201
