# Supplementary material for: Phylogeography, mitochondrial DNA diversity, and demographic history of geladas (Theropithecus gelada)
Source: PLoS One. 2018 Aug 23;13(8):e0202303. doi: 10.1371/journal.pone.0202303 (PMC6107150; doi:10.1371/journal.pone.0202303)
Supplement: S2 Table — (PDF) [file pone.0202303.s004.pdf]

**S2 Table. Best-fit models for protein-coding and non-protein-coding partitions for various gelada populations as obtained from jModeltest for the Bayesian skyline plots (BSPs)**

| population/clade | protein-coding partition | non-protein-coding partition |
|---|---|---|
| global | HKY + I | HKY + I + G |
| central | HKY | HKY + I |
| central-1 | HKY | HKY + I |
| central-2 | F81 | F81 |
| north | HKY + I | HKY + I |
| north-1 | HKY | HKY + I |
| north-2 | HKY | F81 |
| south | F81 | F81 |
