# Supplementary material for: Phylogeography, mitochondrial DNA diversity, and demographic history of geladas (Theropithecus gelada)
Source: PLoS One. 2018 Aug 23;13(8):e0202303. doi: 10.1371/journal.pone.0202303 (PMC6107150; doi:10.1371/journal.pone.0202303)
Supplement: S3 Table — (PDF) [file pone.0202303.s005.pdf]

S3 Table. AMOVA results

number of clades	source of variation	df	ss	variance components	% variation
3	among clades	2	2006.16	23.27	78.38
3	within clades	159	1020.57	6.42	21.62
3	total	161	3026.73	29.69	
F_{ST} = 0.78379					
4	among clades	3	2131.23	24.40	81.11
4	within clades	158	897.93	5.68	18.89
4	total	161	3029.16	30.08	
F_{ST} = 0.81107					
5	among clades	4	2496.59	23.95	86.92
5	within clades	157	565.79	3.60	13.08
5	total	161	3062.38	27.55	
F_{ST} = 0.86921					
6	among clades	5	2523.71	21.86	85.52
6	within clades	156	577.22	3.70	14.48
6	total	161	3100.93	25.56	
F_{ST} = 0.85524					
