# Supplementary material for: Phylogeography, mitochondrial DNA diversity, and demographic history of geladas (Theropithecus gelada)
Source: PLoS One. 2018 Aug 23;13(8):e0202303. doi: 10.1371/journal.pone.0202303 (PMC6107150; doi:10.1371/journal.pone.0202303)
Supplement: S4 Table — (PDF) [file pone.0202303.s006.pdf]

S4 Table. Selection for the "best" population model

number of clades	number of sequences	df	RSS	AIC	delta AIC	BIC	delta BIC	
3	162	2	1020.57	1950.55	91.56	308.34	85.39	
4	162	3	897.93	1931.81	72.82	292.69	69.74	
5	162	4	565.79	1858.98	0	222.95	0	best model
6	162	5	577.22	1864.22	5.24	231.28	8.33	

RSS = error sum of squares (derived from AMOVA)
