# Supplementary material for: Phylogeography, mitochondrial DNA diversity, and demographic history of geladas (Theropithecus gelada)
Source: PLoS One. 2018 Aug 23;13(8):e0202303. doi: 10.1371/journal.pone.0202303 (PMC6107150; doi:10.1371/journal.pone.0202303)
Supplement: S5 Table — (PDF) [file pone.0202303.s007.pdf]

S5 Table. Results of the Automatic Barcode Gap Discovery (ABGD) analyses

Subst model	Pmin/Pmax	X	Partition	Prior intraspecific divergence (P)				
				0.0010	0.0017	0.0028	0.0046	0.0077
JC	0.001/0.1	1.5	Initial	5	5	5	5	1
			Recursive	9	5	5	5	1
K2P	0.001/0.1	1.5	Initial	5	5	5	5	2
			Recursive	9	5	5	5	2
simple	0.001/0.1	1.5	Initial	5	5	5	5	2
			Recursive	5	5	5	5	2
JC	0.001/0.1	1.0	Initial	7	5	5	5	1
			Recursive	11	5	5	5	1
K2P	0.001/0.1	1.0	Initial	12	5	5	5	2
			Recursive	12	5	5	5	2
simple	0.001/0.1	1.0	Initial	5	5	5	5	2
			Recursive	5	5	5	5	2
