# Supplementary material for: Phylogeography, mitochondrial DNA diversity, and demographic history of geladas (Theropithecus gelada)
Source: PLoS One. 2018 Aug 23;13(8):e0202303. doi: 10.1371/journal.pone.0202303 (PMC6107150; doi:10.1371/journal.pone.0202303)
Supplement: S6 Table — (PDF) [file pone.0202303.s008.pdf]

S6 Table. Divergence age estimates (Ma) and respective 95% Highest Posterior Density (HPD) intervals

divergence	div age	95% HPD
Papio – Theropithecus	4.72	4.00-6.21
south + north clades vs central clades	0.67	0.42-0.98
south + north-2 clades vs north-1 clade	0.58	0.35-0.85
south clade vs north-2 clade	0.54	0.31-0.79
central-1 clade vs central-2 clade	0.43	0.25-0.65
first split within south clade	0.03	0.01-0.06
first split within north-1 clade	0.07	0.02-0.12
first split within north-2 clade	0.17	0.09-0.27
first split within central-1 clade	0.20	0.12-0.30
first split within central-2 clade	0.10	0.04-0.17
