# Supplementary material for: Phylogeography, mitochondrial DNA diversity, and demographic history of geladas (Theropithecus gelada)
Source: PLoS One. 2018 Aug 23;13(8):e0202303. doi: 10.1371/journal.pone.0202303 (PMC6107150; doi:10.1371/journal.pone.0202303)
Supplement: S1 Fig — Numbers at nodes refer to bootstrap values in %. (PDF) [file pone.0202303.s001.pdf]

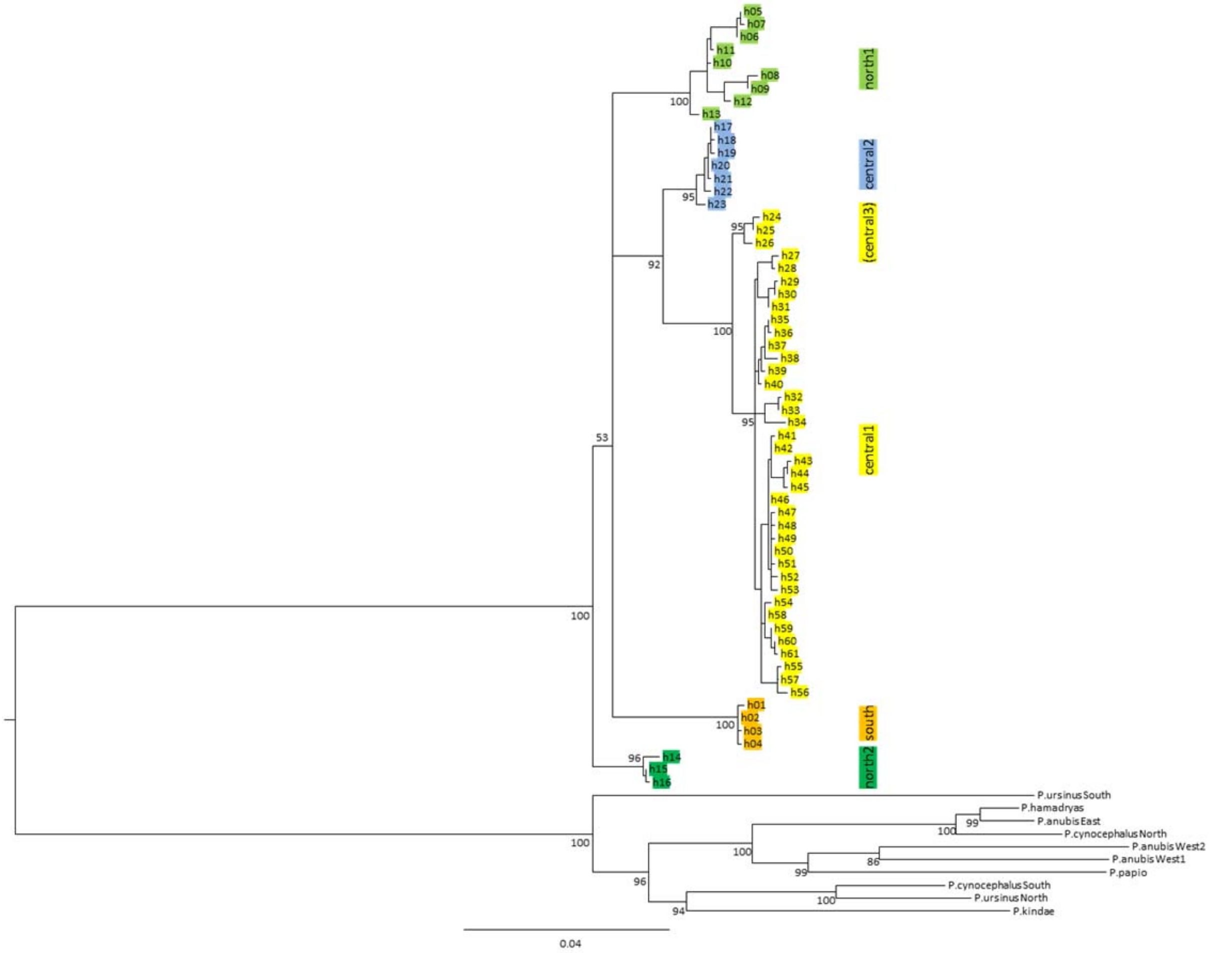

S1 Fig. ML tree showing phylogenetic relationships among gelada and baboon haplotypes. Numbers at nodes refer to bootstrap values in %.
