# Supplementary material for: Phylogeography, mitochondrial DNA diversity, and demographic history of geladas (Theropithecus gelada)
Source: PLoS One. 2018 Aug 23;13(8):e0202303. doi: 10.1371/journal.pone.0202303 (PMC6107150; doi:10.1371/journal.pone.0202303)
Supplement: S1 File — Determining the conservation status of gelada monkeys: Distribution, abundance and phylogenetic relationships of Theropithecus gelada across the Ethiopian Highlands. 2016; Unpubl. Final report to Margot Marsh Biodiversity Foundation. (PDF) [file pone.0202303.s009.pdf]

**S1 File.Nguyen N, Fashing PJ, Burke RJ. Determining the conservation status of gelada monkeys: Distribution, abundance and phylogenetic relationships of *Theropithecus gelada* across the Ethiopian Highlands. 2016; Unpubl. Final report to Margot Marsh Biodiversity Foundation.**

# Determining the conservation status of gelada monkeys: Distribution, abundance & phylogenetic relationships of *Theropithecus gelada* across the Ethiopian Highlands

**2016 Margot Marsh Biodiversity Foundation FINAL REPORT & ADDITIONAL FUNDING REQUEST**

**prepared by**

**Nga Nguyen[1], Peter J. Fashing[1], Ryan J. Burke[2]**
**[1] California State University Fullerton, [2] University of Oxford**

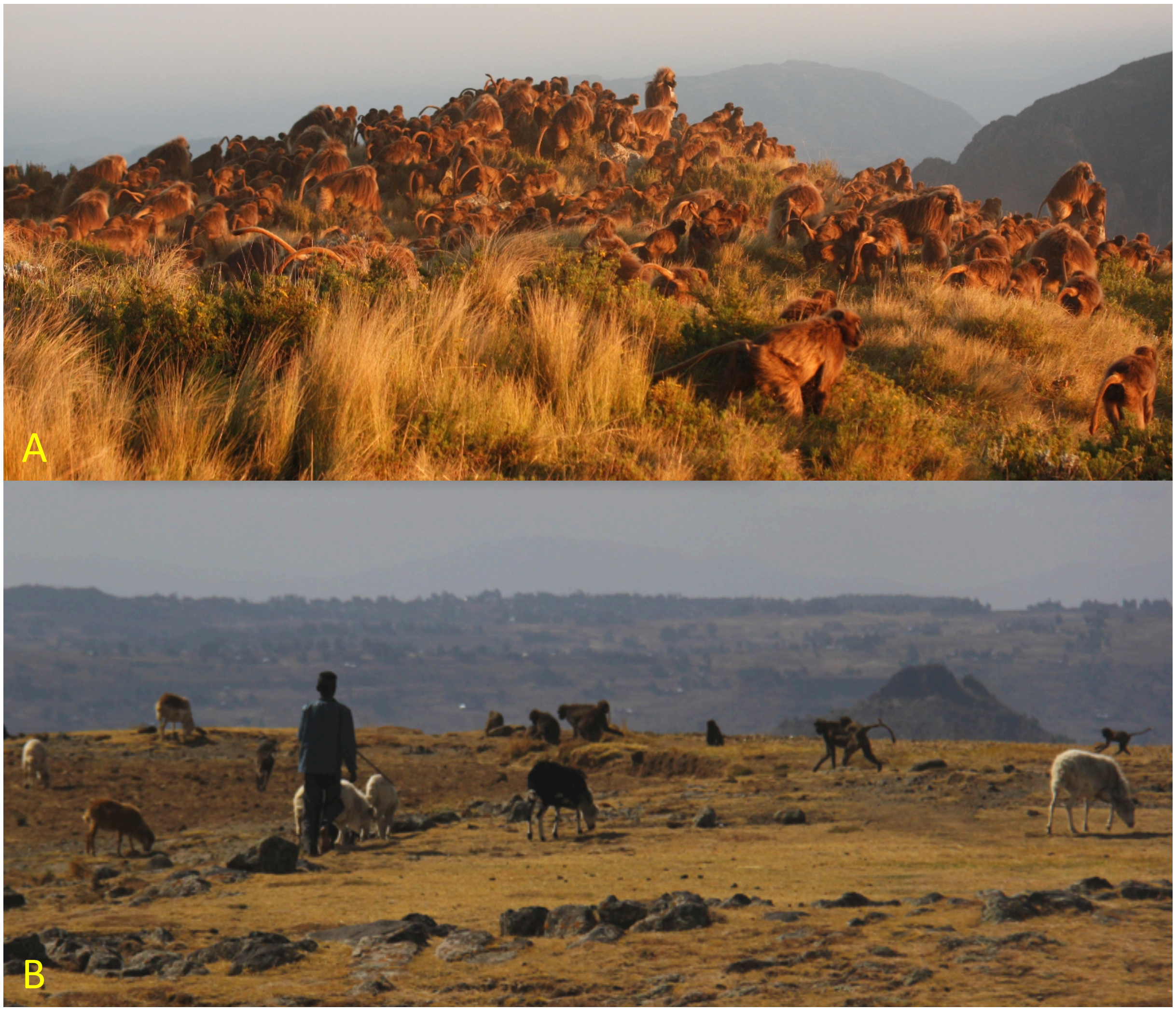


**Fig. 1.** Representative gelada monkey (*Theropithecus gelada*) habitats encountered during recent MMBF-funded surveys for the species across the Ethiopian Highlands. (A) Intact gelada ecosystem on the Guassa Plataeu, Menz escarpment, North Shewa province where geladas form herds of several hundred individuals. (B) Human-dominated, marginal gelada habitat near Tenta, South Wollo province supporting smaller (<40 individuals) gelada herds, living in close proximity to humans, their croplands and rangelands, and domestic livestock.

**SUMMARY**

Gelada monkeys (*Theropithecus gelada*) are the last remaining member of a once speciose genus. Today, geladas are confined to the rapidly shrinking alpine grasslands of the Ethiopian Highlands where they are threatened by conversion of their Afroalpine habitat to crop- and rangeland. Global climate change and a rapidly growing human population in Ethiopia exacerbate these problems. Geladas are currently listed by the IUCN as Not Threatened/Least Concern, even though only 3% of the original Afroalpine habitat remains. Based on our collective experiences studying geladas at three widely separated locations across the species' geographic range during the past decade, we believe the IUCN designation represents a severe underestimate of the current risk faced by geladas. With MMBF support, we carried out systematic censuses for geladas across a large portion (~2/3) of their remaining habitat in the wild in 2015 and 2016. Our objectives in carrying out these censuses were to (1) map the distribution of the species's remaining populations, (2) obtain accurate estimates of the number of geladas in each local population (or deme) as well as obtain an overall estimate of the total population size remaining in the wild, and (3) resolve the phylogeny and taxonomy of geladas at the subspecies level via genetic analyses. Although we are still in the midst of processing and analyzing our field data and samples, our preliminary analyses suggest that while geladas are still found in many parts of their historic range, most remaining populations appear to be small (<40 individuals) and found in highly degraded, marginal habitat, often in close proximity to humans and their livestock. Additional analyses are still underway and promise to yield information vital for assessing the status and distribution of geladas in the wild, as well as contribute to the development of conservation and restoration strategies for the rapidly changing Ethiopian Highlands. Geladas comprise the majority of the native large herbivore biomass throughout the Ethiopian Highlands, and their loss would likely have adverse ecological consequences for people and other animals and plants living in these Highlands. Our ongoing goal is to determine what is left of this once widespread and ecologically important species and what we can learn from the loss or decline of geladas in areas that have already been irreversibly altered by human activities. In addition to providing an update on our results so far, we also wish to request additional funding to complete the final ~1/3 of the surveys to achieve our project objectives in full.

## *INTRODUCTION*

### *Project background & rationale*

The last remaining member of a once widespread and species-rich genus, the gelada (*Theropithecus gelada*) is endemic to the Afroalpine grasslands and adjacent escarpments of the Ethiopian Highlands, occurring at altitudes ranging from 1800 to 4500 m asl (Jablonski 1993). Conversion of gelada habitat to farmland and livestock grazing areas has proceeded at an exponential pace in recent decades and, today, just 3% of the original alpine grassland habitat in the Ethiopian Highlands still exists, making the area one of the fastest disappearing 'biodiversity hotspots' (of 34) on the planet (Williams et al. 2005). Despite immediate and long-term threats posed by global climate change (Dunbar 1998), as well as the growing understanding of the importance of this Eastern Afromontane grassland ecosystem for the maintenance of biodiversity in the region (Williams et al. 2005), geladas continue to be listed as 'Least Concern/Not Threatened' by the IUCN Red List (Gippoliti and Hunter 2008). In our collective opinion, this is a designation that is almost certainly erroneous. Even more fundamentally, both the distributions and the physical characteristics of the two subspecies are poorly defined, and a possible third subspecies has been identified (Gippoliti 2010; Mori & Belay 1990).

Geladas were once regarded as abundant. Indeed, an unsubstantiated estimate placed them at 400,000-600,000 individuals in the 1970s (Dunbar 1977). This estimate continues to plague gelada conservation status because no current quantitative estimates exist. Land use change in the Ethiopian highlands has primarily affected grass and shrubland (Meire et al. 2013). In recent decades, this process has been accelerating, with large tracts of the Afroalpine landscape converted for agriculture and rangeland, a trend likely to continue in the future without intensive management (Funkenberg 2010). Indeed, the human population of Ethiopia has quadrupled in the last century to 100 million, and is projected to reach 180 million people by 2050, making it the tenth most populous country in the world (UNDESA 2013). We believe land use change underlies what our collective, albeit anecdotal, experience studying the three largest remaining populations across the gelada geographic range (northern: Simien Mountains; central: Guassa; southern: Arsi) points to. Our observations suggest gelada numbers were either overestimated in the 1970s or (more likely) have fallen dramatically since then. In particular, our recent censuses of these populations prior to the commencement of the current MMBF-funded study found a combined total of ***less than 6,000 geladas*** [Simien Mountains: ~4250 (Beehner et al. 2007), Guassa: ~810 geladas (Fashing & Nguyen, unpub. data), Arsi: ~600 geladas (Mekonnen, unpub. data)]. ***These numbers produce an extrapolated estimate that is several orders of magnitude lower than the 1970s estimate and creates considerable cause for concern.***

### *Are geladas a keystone species?*

Large mammals have been widely recognized to play key roles in ecosystem functioning (McNaughton et al 1997). Through habitat engineering and interactions with diverse constituents of the faunal and floral communities, animal control of physiochemical processes and energy flow reverberates through the ecosystem (Jones et al. 1994). Species defined as "keystones" exhibit a disproportionately large control over the structure and functioning of ecosystems relative to their abundance (McNaughton et al 1997).

Geladas are grazers that obtain ~90% of their diet from grass blades in the rainy season when their nutritional value is high, but shift their diet to herbs, seeds and underground plant parts (i.e. rhizomes) in the dry season (Dunbar 1977; Fashing et al. 2014). Their habitat is characterized by a diversity of plant community types; matrices of 'guassa' tussock grasses (*Festuca* spp.), short herbs (*Alchemilla* spp.) and grasses, giant lobelias (*Lobelia rhynchopetalum*) and Ericaceous moorlands (*Erica and Phillipia* spp.) (Fashing et al. 2014). Erosion of their ancient Basaltic plateau habitats has been occurring for millennia, leading to the evolution of a plant community structure that is of vital importance to the retention of soil (Meire et al 2013). The region is susceptible to high inter- and intra-annual variation in precipitation, often experiencing a prolonged and intense rainy season from June to September but also being very vulnerable to drought (Nyssen et al 2005). Although there has been much research into the impact of cropland and livestock grazing on erosion and other ecosystem processes in Ethiopia (Descheemaeker et al. 2006; Billi 2015), there has been very little attention paid to the influence of native grazer species on these processes. Carrying out a sorely needed census of *T. gelada* to illuminate their current conservation status provides an ideal opportunity to test their keystone role in the Afroalpine system through a spatially explicit comparison of animal, plant and soil landscape patterns and processes.

***Project objectives***

Because our preliminary evidence suggests that geladas are far less common than is currently recognized, the primary objective of this study is to carry out a comprehensive countrywide census – which is now 2/3 complete – for geladas that will identify (1) **how many gelada populations remain**, and (2) **the total number of geladas in each population**. Because gelada phylogeny and taxonomy still remains unresolved (Gippoliti 2010), a 3rd objective of our study focused on biogeography and isolation, specifically the use of phylogenetic analyses to determine **genetic variation within and between the remaining gelada populations**. The data we are collecting from the censuses and subsequent genetic analyses will be essential to determining an accurate conservation status of geladas as a species as well as the respective status of each of the subspecies (two are currently recognized, though genetic analyses may reveal that there are more, Gippoliti 2010). We anticipate our surveys will not only impact the status of geladas on the IUCN Red List, but also inform conservation and management policies for geladas within Ethiopia. In particular, the genetic data will be crucial in understanding barriers between local populations (between supposed subspecies ranges), and from north and south of the Rift Valley. Although the Ethiopian government and Ethiopian Wildlife Conservation Authority are eager to know how many geladas remain, Ethiopia is one of the world's poorest countries and funds are not available internally to support such countrywide surveys. We are confident that we will be able to resolve the status of Ethiopia's flagship primate species and make important contributions towards its conservation. This report lays out the progress we have made thus far.

## METHODS

### *Study areas*

We searched the published literature and historical records and identified 11 sites where geladas are known to occur or where they have been reported to occur in the past (Fig. 2). These sites are scattered primarily across the central and northern Ethiopian Highlands and encompass Shewa, Wollo, Gonder, Gojjam, Tigray and Oromia regions, an area covering ~51,000 km$^2$. The habitat types found in these areas today include intact Afroalpine grasslands in protected areas, but also fragmented and isolated suitable gelada habitat outside protected areas in human dominated landscapes.

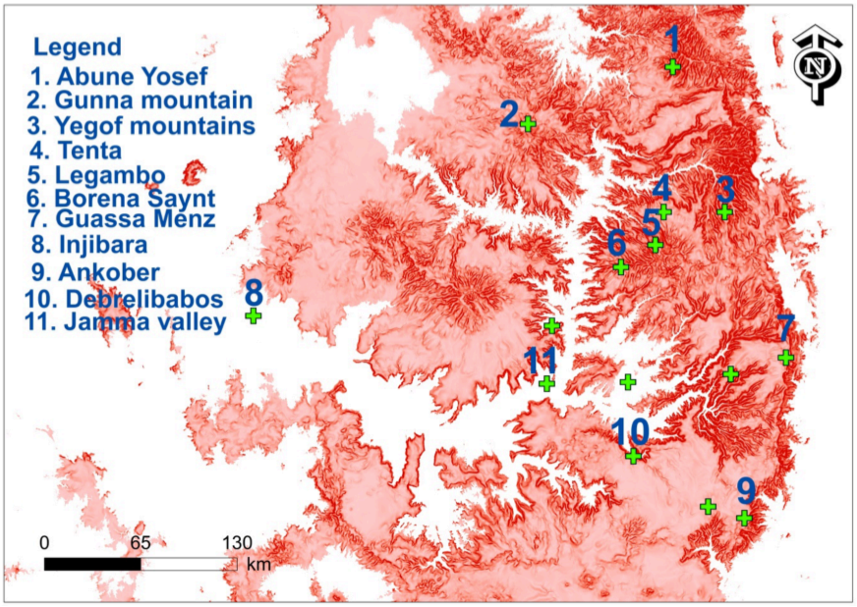


**Fig. 2.** Sites where geladas have been recorded (pre-2014). + = confirmed gelada populations. Slope values in degrees range from 0-90, with 90 indicating a vertical cliff. The range of slope values is 0-69.

To carry out our censuses, we first identified potentially suitable gelada habitat using computer modeling. We derived a simple model predicting gelada suitable habitat from a Digital Elevation Model (DEM) and broad vegetation zones. For 320 existing gelada presence locations, 99% were located in the predicted suitable habitat (defined as areas above 1800m asl and within 2km of a cliff, with >20˚ slope, Fig. 3). The highly specialized habitat

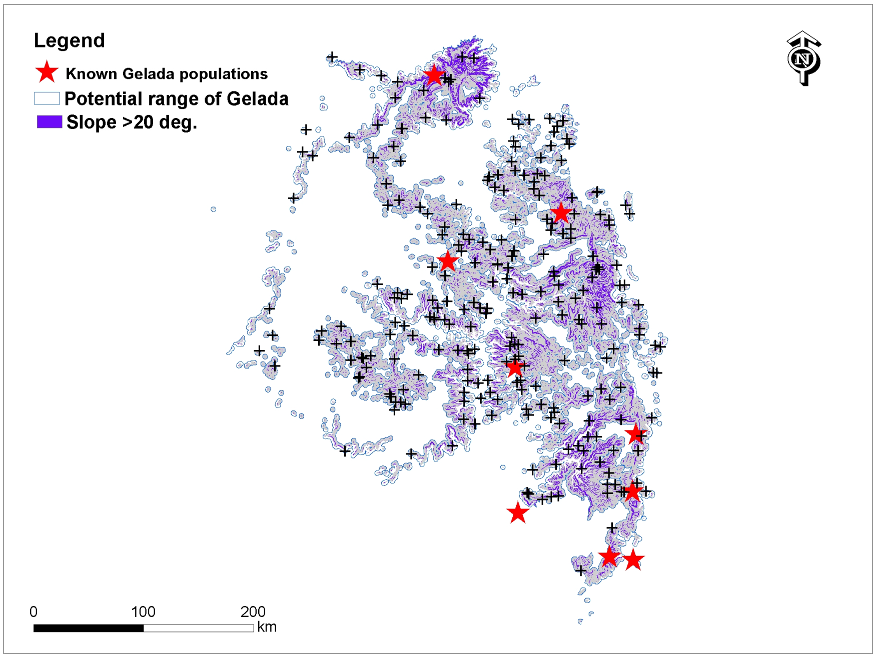


**Figure 3.** Areas of potential or known gelada occupancy. In the figure, + represents randomly chosen survey locations within the species's potential suitable habitat range (2 km from cliff; slope >20˚; Amhara region only)

requirements of geladas (grasslands above 1,800 m and within 2-3 km of a near-vertical rock face that serves as a sleeping cliff and retreat from predators) means that it has been relatively simple to narrow down the list of potential locations where geladas might still occur, simplifying transect selection. Promising survey localities (n=1124 different locations on different cliffsides) were then chosen at random within the areas of known or potential gelada occupancy derived from the suitable habitat model (Fig. 3).

***Abundance and population size of geladas***

Base camps were established at each survey site to enable observers to sample the region as comprehensively and effectively as possible, usually in less than 7 days. Systematic censuses were carried out via the 'direct count method' which is optimal for censusing a species like geladas, which live in mountainous but very open terrain (Beehner et al, 2007). Sweep censuses were used with 3 to 6 observers at a time, to determine population size and density of geladas. Observers simultaneously walked along transects 2-10 km in length, as time allowed, along the cliff-faces (Whitesides et al, 1988). The observers walked along the transect at an average speed of 1-2 km/h on foot depending on the habitat, usually starting in the mid-morning when gelada groups tend to leave their sleeping cliffs for foraging (Hunter, 2001). We ensured the boundaries of a single day's census did not overlap that of the subsequent day's census so as to avoid double-counting on the following census day. During censuses, we also conducted sweep censuses of all other large herbivores, native and domestic, according to the same protocol. Over the course of 3 days in both Simien Mountains National Park and Borena Saynt National Park, we collaborated with National Park and research staff there to survey with up to 15 observers simultaneously in these unusually large survey areas.

***Genetic structure and intraspecific divergence***

Faecal samples were collected noninvasively from geladas at each census site for later genetic analyses. At each locality, observers attempted to sample a target of 5% of the population. Samples were preserved in 30 ml tubes containing >97% ethanol at least for 24 hours. After 24 hours, we decanted the ethanol and left the tube open for some time for the sample to dry. Then, the sample was transferred to a new test tube containing silica beads separated by small pieces of tissue paper to avoid direct contact of the feces with silica. In addition, tissue samples were collected opportunistically from dead specimens. Data recorded for each sample collected include the date, name of collector, species, identification/code number of the sample, GPS location, altitude, age and sex of the individual (if known), condition of feces collected, habitat type, group size and other features. The samples were stored in the field for several few weeks before being transported to Addis Ababa University and stored under -4C until they cam be transported to the German Primate Centre (DPZ), Germany for further genetic analyses. Zinner & Roos (collaborators on this project) have secured funding from Primate Action Fund (Zinner & Roos, PAF 13-14) to carry out the genetic analyses.

***Gastrointestinal parasite infections***

Faecal samples were also collected noninvasively at each site for later laboratory analysis for gelada gastrointestinal parasites. Samples were collected immediately after defecation from the

inner part of the feces to avoid contamination (Gillespie, 2006). As before, a target of fecal samples from 5% of each potential gelada population/deme or survey site was aimed for to assess the potential parasitic infection across sites. Before collecting fecal samples, it was necessary to examine the sample macroscopically for the presence of blood, mucus, tapeworm proglottids and adult or larval nematods (Gillespie, 2006). Replicate fecal samples (ca. 3g) were stored in a 15 ml sterile plastic vial with 7-8 ml of 10% formalin solution and ≥99.5 % ethanol. The same attributes were recorded as the genetic samples (above). In addition, any external parasitic infection and swelling observed on geladas were recorded including the age/sex class of individuals and other associated features recorded above. The samples were transported to the Addis Ababa University, Department of Zoological Sciences where they are being preserved at 4°C or below.

***Vegetation composition and biogeochemistry***

The plant community composition at each survey site was examined through rapid botanical assessments of randomly selected vegetation plots on survey transects. We recorded species diversity and relative biomass. All census participants were trained in basic botanical identification and soil/vegetation sampling, and equipped with a GPS unit and digital camera. All participants attempted to reach a target of five plots per hour, with at least 20 m distance separating points, ensuring an equal coverage in all six main habitat types: *Festuca* grassland, *Euryops-Alchemilla* shrubland, Mima mounds (*Euryops-Festuca* grassland), Erica moorland, *Helichrysum-Festuca* grassland, and swamp grassland (Ashenafi et al, 2012). Habitat classification will be defined for all survey locations on the basis of these plots, as well as regular ground control points and unmanned aerial vehicle (UAV) imagery. Image classification will be performed by using a moving window approach at a 5 x 5 m resolution, which can estimate the proportion of the vegetation community of the three most abundant plants. Site characteristics – e.g., stone cover and topography, soil moisture – were recorded using a WET-sensor (Delta-T Device), and samples of above-ground biomass and surface soils. We will create Digital Terrain Models (DTMs) and ortho-image mosaics after creating photogrammetric image blocks by bundle-block triangulation from georeferenced photo log files (Linder 2009). The resulting fine-scale mosaics (approx. 10 cm) and DTMs will be used to regress the plot vegetation and nutrient data to the larger spatial extent (Fig. 4).

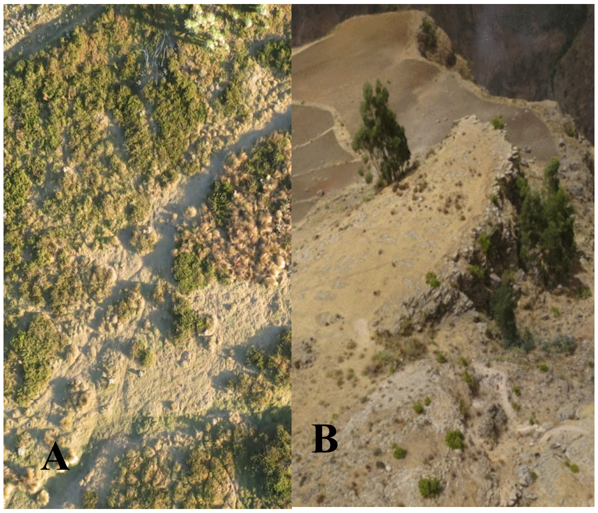

**Figure 4.** Example UAV-derived imagery; (A) Guassa (Community Conservation Area, in N. Shewa; *Festuca* grassland and grazing lawn mosaic); (B) Were Ilu region (no protection, in S. Wollo; high percentage of bare ground)

## PRELIMINARY RESULTS

***(1) How many geladas are left and where are they?*** To date, we have surveyed for geladas across much of their historical range, in North and South Shewa, East Gojjam, North Gondar, North and South Wollo, and Tigray (Fig. 6). All censuses were undertaken by a team of 4-5 observers trained and usually accompanied by RJB), first in 2015 (March-July) and again in 2016 (January-June). In total, we have collectively covered >22,000 km by road and systematically walked ~1,235 km of cliffside transects. Our analyses of the survey data are still underway, but to date, our preliminary analyses suggest that the vast majority (>80%) of gelada populations surveyed thus far comprise fewer than 40 individuals, significantly less than populations inhabiting more ecologically intact ecosystems such Borena Sayint NP (>300 geladas) and Guassa-Menz Community Conservation Area (>800 geladas) (Fig. 5). Nearly all of the marginal populations surveyed thus far occupy degraded Afroalpine grassland habitats characterized by low plant diversity and biomass.

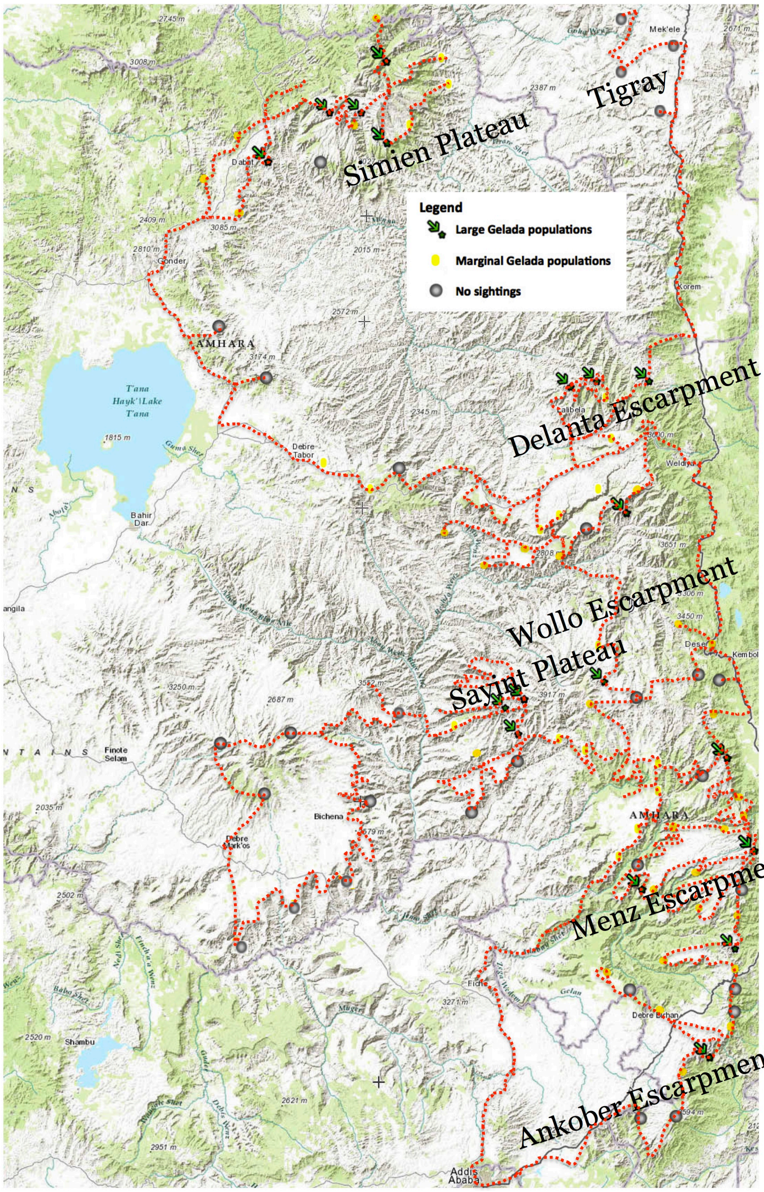


**Figure 5.** Major *T. gelada* census sites in 2015 and 2016, categorized as large (>40 animals), marginal (<40), or absent. Faecal (phylogenentic, parasitological), botanical and soil samples were collected from each site.

To date, we have carried out transects at nearly 2/3 of the sites identified in our suitable habitat model as potentially capable of supporting geladas in the Ethiopian Highlands (n=724 of the 1124 randomly generated cliffside transect sites from our habitat suitability model). We are currently analyzing the data and samples collected from these surveys and finalizing habitat and demographic modeling to identify those sites that require a revisit for land cover and/or census purposes, as well as high priority sites that

have not been visited yet. We are hopeful that more geladas will be found in the remaining survey areas, which will focus on sections of Tigray, North Wollo, Delanta and Gonder regions in the north and Arsi in the south (Fig. 6). These areas may contain vestiges of the gelada populations that once were commonly seen across the Ethiopian Highlands, and living in more ecologically intact environments than their counterparts elsewhere in the country, but only by visiting these sites and surveying for geladas at these sites can we determine with any degree of certainty the true conservation status and distribution of this flagship monkey species for the rapidly disappearing Ethiopian Highlands ecosystem.

***(2) Genetic structure within and across gelada populations.*** To date, we have collected 367 fecal samples for genetic (and parasitological) analyses. While the laboratory analyses are still underway, our preliminary genetic analyses suggest three distinct gelada haplotype groupings are distributed across the species's current range.

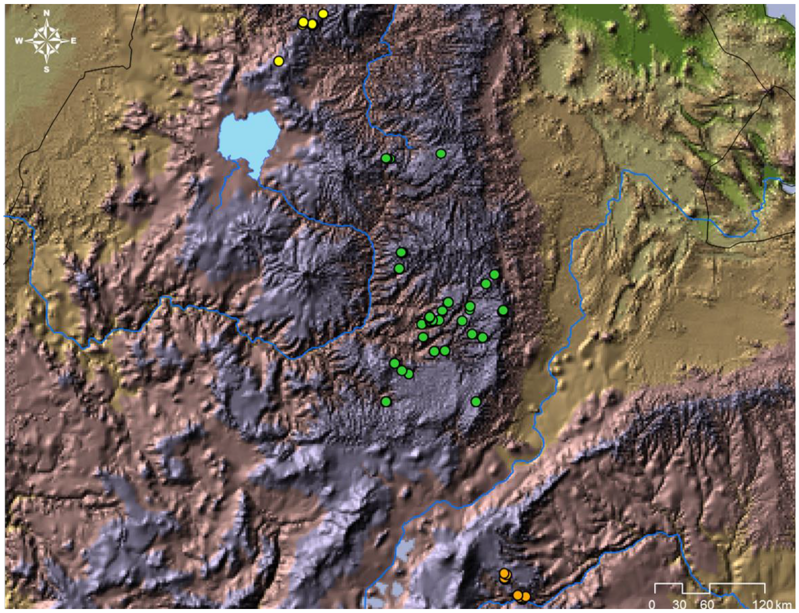


**Figure 6.** Geographic distribution of resulting haplotypes from the northern and central parts of Ethiopia. Adapted from a map provided by Dietmar Zinner (DPZ). Dots indicate sample locations in the species range of *T. gelada*. yellow = northern haplotypes; green = central haplotypes; orange = southern haplotypes. *(2015 only)*

***(3) Survey site plant and soil characteristics.*** We established and evaluated 1270, 4x4m botanical plots across the different survey sites. In ½ of these plots (635), we took soil and plant samples. To date, we have processed 412 of these plots for soil bulk density, volumetric water content, organic matter content, and soil C and N concentrations. Plant and soil subsamples are being analysed for $^{15}N$:$^{14}N$ ratios (standardized relative to air, d$^{15}$N), as well as N, Carbon (C), and Phosphorous (P) concentrations. Our continuing analyses of these samples and data will enable us to identify those plant community characteristics and biogeochemical features of sites supporting thriving gelada populations from those supporting marginal (or no) gelada populations.

Below, we append a copy of our project's timeline (**Appendix 1**), an accounting of how the previous MMBF award was disbursed (**Table 1**), a request for additional funding and proposed budget (**Table 2**).

**PROJECT COLLABORATORS**

Dr. Anagaw Atickem, Ethiopian national
Postdoctoral Researcher, Department of Biological Science, University of Oslo, Norway

Dr. Afework Bekele, Ethiopian national
Professor, Department of Biology, Addis Ababa University, Addis Ababa, Ethiopia

Dr. Jacinta Beehner, American national
Associate Professor, Department of Anthropology, University of Michigan, U.S.A.

Dr. Thore Bergman, American national
Associate Professor, Department of Anthropology, University of Michigan, U.S.A.

Ryan Burke, Canadian National
Ph.D. Student, Department of Zoology, University of Oxford, UK

Dr. Peter Fashing, American national
Associate Professor, Department of Anthropology, California State University Fullerton,,U.S.A.

Addisu Mekonnen, Ethiopian national
Ph.D. Student, Department of Biological Science, University of Oslo, Norway;
Lecturer, Department of Biology, Addis Ababa University, Ethiopia

Amera Moges, Ethiopian national
Ph.D. Student, Department of Biology, Addis Ababa University, Ethiopia

Dr. Nga Nguyen, American national
Associate Professor, Department of Anthropology, California State University Fullerton, U.S.A.

Dr. Christian Roos, German national
Director, Gene Bank of Primates and Primate Genetics Laboratory, German Primate Center (DPZ), Göttingen, Germany

Dr. Nils Chr. Stenseth, Norwegian national
Professor and Director, Centre for Ecological & Evolutionary Synthesis, University of Oslo, Oslo, Norway

Dr. Dietmar Zinner, German national
Senior Scientist, Department of Cognitive Ethology, German Primate Center (DPZ), Göttingen, Germany

**ACKNOLWEDGMENTS**

We thank the Margot Marsh Biodiversity Foundation and the British Ecological Society for their generous support of our gelada surveys across Ethiopia as well as San Diego Zoo for supporting our long-term, intensive research on geladas at Guassa, Ethiopia. We are extremely grateful to the Department of Zoological Sciences, Addis Ababa University, and staff and students of the Guassa Gelada Research Project, Bale Monkey Research Project, Ethiopian Wolf Conservation Programme, and Simien Gelada Research Project for their logistical support of this project. We are also very thankful to Claudio Sillero, Jorgelina Marino, Hans Bauer, Eric Bedin, and Girma Eshete for their feedback, assistance, and/or sharing of data. We also thank the Ethiopian Wildlife Conservation Authority, and government officials in the following provinces/regions: North and South Shewa, North Gondar, North and South Wollo Zones, East Gojjam, Tigray and Oromia for their permissions to conduct this research. This study would not have been possible without the assistance of several research assistants (Mengistu Birhan, Firde Sultan, Zemenu Birhan, Yenegeta Bayhe, Amare Mezgebu, and Mamar Dilnesa), PhD students (Dereje Yazezew – Debre Birhan University, Hussein Ibrahim – Addis Ababa University, and Zewdu Kifle – Bahir Dar University), field camp attendants (Shoafera Tessema and Badiloo Muluyee), and drivers (Tamiru, Elias, Getnet and Shatu). Lastly, we thank the local district (*kebele* and *woreda*) officials and residents in the survey areas for their invaluable support of and assistance with this project.

## REFERENCES


Ashenafi ZT, Leader-Williams N, & Coulson T. 2012. Consequences of human land use for an Afroalpine ecological community in Ethiopia. *Conservation and Society* 10: 209.

Asner, GP, Vaughn N, Smit IPJ, & Levick, S. 2016. Ecosystem-scale effects of megafauna in African savannas. *Ecography* 39: 240–252.

Bakker ES, Olff H, Boekhoff M, Gleichman JM, & Berendse F. 2004. Impact of herbivores on nitrogen cycling: contrasting effects of small and large species. *Oecologia* 138:91-101.

Beehner JC, Gebre B, Bergman TJ, & McCann C. 2007. Population estimate for geladas (*Theropithecus gelada*) living in and around the Simien Mountains National Park, Ethiopia. *SINET Ethiopian Journal of Science* 30: 149-154.

Billi P. 2015. *Landscapes and landforms of Ethiopia*. Springer, Dordrecht, Netherlands.

Craine JM, Ballantyne F, Peel M, Zambatis N, Morrow C, & Stock WD. 2009. Grazing and landscape controls on nitrogen availability across 330 South African savanna sites. *Austral Ecology* 34: 731-740.

Davidson AD. 2005. The comparative and interactive effects of prairie dogs and banner-tailed kangaroo rats on plants and animals in the northern Chihuahuan Desert. Ph.D. thesis, Univ. of New Mexico.

Day TA & JK Detling. 1990. Grassland patch dynamics and herbivore grazing preference following urine deposition. *Ecology* 71: 180–188.

Delibes-Mateos M, Delibes M, Ferreras P, & Villafuerte R. 2006. Key role of European rabbits in the conservation of the western Mediterranean basin hotspot. *Conservation Biology* 22: 1106-1117.

Descheemaeker K, Nyssen J, Rossi J, Poesen J, Haile M, Raes D, Muys B, Moeyersons J, & Deckers S. 2006. Sediment deposition and pedogenesis in exclosures in the Tigray Highlands, Ethiopia. *Geoderma* 132: 291-314.

Dunbar RIM. 1977. The gelada baboon: status and conservation. IN: Rainier LHMBG, and Bourne GH, editors. *Primate Conservation*. New York: Academic Press, pp. 363-383.

Dunbar RIM. 1998. Impact of global warming on the distribution and survival of the gelada baboon: a modeling approach. *Global Change Biology* 4: 293-304.

Fashing PJ, Nguyen N, Kerby JT, Venkataraman VV. 2014. Gelada feeding ecology in an intact ecosystem at Guassa, Ethiopia: Variability over time and implications for theropith and hominin dietary evolution. *American Journal of Physical Anthropology* 155:1-16.

Frank DA, Groffman PM, Evans RD, Tracy BF. 2000. Ungulate stimulation of nitrogen cycling and retention in Yellowstone Park grasslands. *Oecologia* 123:116–121.

Funkenberg, T. 2010. Remote sensing based assessment of land-cover change in the Afroalpine ecosystem of North Wollo (Ethiopia). M.Sc. Thesis, Trier University, Trier, Germany.

Fynn RW & Bonyongo MC 2011. Functional conservation areas and the future of Africa's wildlife. *African Journal of Ecology* 49:175-188.

Gillespie, T.G. 2006. Noninvasive assessment of gastrointestinal parasite infections in free-ranging primates. *International Journal of Primatology* 27: 1129-1143.

Gippoliti S. 2010. *Theropithecus gelada* distribution and variations related to taxonomy: history, challenges and implication for conservation. *Primates* 51(4):291-297.

Gippoliti S, and Hunter CP. 2008. *Theropithecus gelada*. IUCN Red List of Threatened Species Version 20131.

Hunter, CP. 2001. Ecological determinants of gelada (*Theropithecus gelada*) ranging patterns. PhD thesis, University of Liverpool, Liverpool, U.K.

Jablonski NG, editor. 1993. *Theropithecus: The rise and fall of a primate genus*. Cambridge: Cambridge University Press.

Jones CG, Lawton JH, and Shachack, M. 1994. Organisms as ecosystem engineers. *Oikos* 69:373-386.

Kotliar NB. 2000. Application of the new keystone-species concept to prairie dogs: how well does it work? *Conservation Biology* 14:1715-1721.

Linder, I.W. 2009. *Digital photogrammetry*. Springer, Berlin.

Marino J, Sillero-Zubiri C & Macdonald DW. 2006. Trends, dynamics and resilience of an Ethiopian wolf population. *Animal Conservation* 9:49-58.

McNaughton SJ, Banyikwa FF, McNaughton MM. 1997. Promotion of the cycling of diet-enhancing nutrients by African grazers. *Science* 278:1798-1800.

Meire E, Frankl A, Wulf AD, Haile M, Deckers J, Nyssen J. 2013. Land use and cover dynamics in Africa since the nineteenth century: warped terrestrial photographs of North Ethiopia. *Regional Environmental Change* 13:717-737.

Mittermeier RA, Gil PR, Hoffman M, Pilgrim J, Brooks T, Goettsch Mittermeier C, Lamoureux J, and da Fonseca GAB. 2005. *Hotspots revisited: Earth's biologically richest and most endangered terrestrial ecoregions*. Washington, D.C.: Conservation International.

Mittermeier RA, Turner WR, Larsen FW, Brooks TM, Gascon C. 2011. Global biodiversity conservation: the critical role of hotspots. IN: *Biodiversity hotspots*, eds. Zachos FE & Habel JC, Springer, Berlin, pp. 3-22.

Mori, A. and Gurja Belay 1990. The distribution of baboon species and a new population of Gelada baboons along the Wabi-Shebeli River, Ethiopia. *Primates* 31:495–508.

Nyssen J, Vandenreyken H, Poesen J, Moeyersons J, Deckers J, Haile M, Salles C, Govers G. 2005. Rainfall erosivity and variability in the northern Ethiopian highlands. *Journal of Hydrology* 311: 172-187.

Olff H, Vera FWM, Bokdam J, Bakker ES, Gleichman JM, Maeyer KD, & Smit R. 1999. Shifting mosaics in woodlands driven by the alternation of plant facilitation and competition. *Plant Biology* 1: 127-137.

Pastor, JR. Moen A., and Y. Cohen. 1997. Spatial heterogeneities, carrying capacity, and feedbacks in animal– landscape interactions. *Journal of Mammalogy* 78:1040– 1052.

Tilman, D.; May, R. M.; Lehman, C. L.; Nowak, M. A. 1994. Habitat destruction and the extinction debt. *Nature* 371:65-66

United Nations Department of Economic and Social Affairs, Population Division. (2013). World population prospects: The 2012 revision. Key findings and advance tables. Working paper no. ESA/P/WP.227, New York.

Williams S, Pol JLV, Sprawls S, Shimelis A, and Kelbessa E. 2005. Ethiopian Highlands. IN: Mittermeier RA, Gil PR, Hoffman M, Pilgrim J, Brooks T, Goettsch Mittermeier C, Lamoureux J, and da Fonseca GAB. 2005. *Hotspots Revisited: Earth's biologically richest and most endangered terrestrial ecoregions*. Washington, D.C.: Conservation International.

Whitesides GH, Oates, JF, Green SM & Kluberdanz RP. 1988. Estimating primate densities from transects in a West African rain forest: a comparison of techniques. *Journal of Animal Ecology* 345-367.

Zinner, D. and Roos C. 2013-14. Primate Action Fund grant to study gelada phylogenetics. Conservation International.

Zinner D, and Torkler F. 1996. GIS and remote sensing techniques as tools for surveying primates. *Ecotropica* 2:41-47.

**APPENDIX 1. Project Timeline**

a) January-November 2015: Census team surveyed potential gelada sites in the Northern Highlands. Genetic work on the faecal samples began in Germany in Roos and Zinner's German Primate Center genetics lab.
b) May 2015: Census team visited Simien Mountains where Beehner and Bergman helped coordinate survey work at this (their long-term behavioral research) site and its environs, which contains by far the largest remaining gelada population on record. Other sites across the Northern Highlands were also surveyed and faecal samples were also collected at most sites (Figures 6&7).
c) January-June 2016: Census team surveyed several additional potential gelada sites in the Northern Highlands.
d) July-December 2016: Habitat Classification and production of 3D orthomosaics by Burke for all survey sites using Agisoft Photoscan Professional (0.85) software, and ERDAS Imagine software. Image classification is performed by using a moving window approach to at a 5 x 5 m resolution from the Red, Green, and Blue light bands to define habitat "classes".
e) **July 2016-March 2017**: Data analyses from population censuses with GIS mapping of gelada distribution ongoing. Population density is estimated using DISTANCE (Version 5.0, Plumptre & Cox, 2006). Other analyses, including those of the faecal samples collected in the field, also ongoing.
f) **January - March 2017**: Census team will survey final 1/3 of sites identified as potentially capable of supporting remaining populations of geladas in the Ethiopian Highlands.
g) **March – December 2017**: Prepare peer-reviewed scientific publications on (1) gelada distribution and abundance across Ethiopia [1st author will be Mekonnen], (2) gelada phylogeny and evolutionary history [1st author will be Zinner], and (3) gelada and domestic herbivore impact on plant communities and ecosystem processes [1st author will be Burke]. Produce reports for the Ethiopian government and Ethiopian Wildlife Conservation Authority (EWCA) containing the results of our research and our recommendations for the best strategies for the conservation of geladas and their rapidly disappearing Afroalpine habitat. Petition IUCN to change the status of geladas on their Red List, pending the results of our surveys and molecular taxonomic research. Develop and promote landscape restoration plans at ~10 sites across the Shewa, Wollo, Gonder and Tigray regions which would improve connectivity between gelada populations.

**Table 1. BUDGET – MMBF Funds Already Spent**

We thank the MMBF fund for their generous contribution of $15,000 (USD) to this scientific endeavor thus far. The accomplishments described in this report, and the data and samples collected and undergoing careful analysis by an international team of researchers (from Canada, Ethiopia, Norway, England, Germany and the USA) would not have been possible without the support of the MMBF. Below, we outline the items on which we spent the MMBF funds.
